# Methods for isolation and transcriptional profiling of individual cells from the human heart

**DOI:** 10.1016/j.heliyon.2020.e05810

**Published:** 2020-12-29

**Authors:** Neha Pimpalwar, Tomasz Czuba, Maya Landenhed Smith, Johan Nilsson, Olof Gidlöf, J. Gustav Smith

**Affiliations:** a Department of Cardiology, Clinical Sciences, Lund University, Lund, Sweden; b Department of Cardiothoracic Surgery, Sahlgrenska University Hospital, Gothenburg, Sweden; c Department of Molecular and Clinical Medicine, Institute of Medicine, Gothenburg University, Gothenburg, Sweden; d Department of Cardiothoracic Surgery, Clinical Sciences, Lund University and Skåne University Hospital, Lund, Sweden; e Department of Heart Failure and Valvular Disease, Skåne University Hospital, Lund, Sweden; f Wallenberg Center for Molecular Medicine and Lund University Diabetes Center, Lund University, Lund, Sweden; g Department of Cardiology, Sahlgrenska University Hospital, Gothenburg, Sweden

**Keywords:** Human, Heart, Single cell, Methods, Protocol, Transcriptomics, Transcriptome, Health sciences, Cardiology, Cardiovascular System

## Abstract

**Background:**

Global transcriptional profiling of individual cells represents a powerful approach to systematically survey contributions from cell-specific molecular phenotypes to human disease states but requires tissue-specific protocols. Here we sought to comprehensively evaluate protocols for single cell isolation and transcriptional profiling from heart tissue, focusing particularly on frozen tissue which is necessary for study of human hearts at scale.

**Methods and results:**

Using flow cytometry and high-content screening, we found that enzymatic dissociation of fresh murine heart tissue resulted in a sufficient yield of intact cells while for frozen murine or human heart resulted in low-quality cell suspensions across a range of protocols. These findings were consistent across enzymatic digestion protocols and whether samples were snap-frozen or treated with RNA-stabilizing agents before freezing. In contrast, we show that isolation of cardiac nuclei from frozen hearts results in a high yield of intact nuclei, and leverage expression arrays to show that nuclear transcriptomes reliably represent the cytoplasmic and whole-cell transcriptomes of the major cardiac cell types. Furthermore, coupling of nuclear isolation to PCM1-gated flow cytometry facilitated specific cardiomyocyte depletion, expanding resolution of the cardiac transcriptome beyond bulk tissue transcriptomes which were most strongly correlated with PCM1^+^ transcriptomes (r = 0.8). We applied these methods to generate a transcriptional catalogue of human cardiac cells by droplet-based RNA-sequencing of 8,460 nuclei from which cellular identities were inferred. Reproducibility of identified clusters was confirmed in an independent biopsy (4,760 additional PCM1^-^ nuclei) from the same human heart.

**Conclusion:**

Our results confirm the validity of single-nucleus but not single-cell isolation for transcriptional profiling of individual cells from frozen heart tissue, and establishes PCM1-gating as an efficient tool for cardiomyocyte depletion. In addition, our results provide a perspective of cell types inferred from single-nucleus transcriptomes that are present in an adult human heart.

## Teaser

Human; Heart; Single cell; Transcriptomics; Methods; Protocol; Transcriptome; Health sciences; Cardiology; Cardiovascular System

## 1 Introduction

Single-cell RNA-sequencing (scRNA-seq) allows quantitative analysis of the transcriptomes of individual cells from tissues, across dynamic states and functional processes (Regev et al., 2017). Current technologies allow sequencing of thousands of cells per experiment, and when applied to several human tissues such as brain (Habib et al., 2017; Lake et al., 2016), liver (MacParland et al., 2018), kidney (Lake et al., 2019), lung (Vieira Braga et al., 2019), gut (Smillie et al., 2019), bone marrow (Oetjen et al., 2018), and circulating leukocytes (Villani et al., 2017) have allowed identification of novel cell types and improved description of molecular transitions under dynamic conditions such as during organ development (Asp et al., 2019; Cui et al., 2019; DeLaughter et al., 2016). The different protocols used in such studies underscore the need for tissue-specific protocols to generate single cell suspensions (Lafzi et al., 2018; Luecken and Theis, 2019; Regev et al., 2017). To our knowledge, although the first protocol for scRNA-seq was published in 2009 (Tang et al., 2009) no study has yet comprehensively evaluated protocols for single-cell isolation and scRNA-seq of adult human heart tissue, although several scRNA-seq studies have examined murine hearts (Ackers-Johnson et al., 2018; Burke et al., 2016; Farbehi et al., 2019; Gladka et al., 2018; Molenaar and van Rooij, 2018; Skelly et al., 2018) and a few recent studies have examined human hearts (See et al., 2017, Wang et al., 2020; Tucker et al., 2020; Litviňuková et al., 2020). Such information could be of substantial value, given the current role of heart disease as leading cause of death globally and the pathophysiological heterogeneity of conditions with adverse effects on the heart. Importantly, beyond cardiomyocytes, the human heart contains several other cell types which are central in cardiac damage responses, remodelling, and disease. Traditional methods for study of the cardiac transcriptome have focused on bulk tissue, which is heavily biased towards cardiomyocyte expression, or have relied on culturing which is likely to introduce major molecular perturbations. Further, the fibrous nature of human cardiac tissue makes enzymatic dissociation challenging, which is further complicated by the necessity for use of frozen rather than fresh tissues given the difficulty of obtaining viable human heart tissue. Thus, we sought to comprehensively evaluate protocols for cardiac single-cell isolation to allow high-quality scRNA-seq analysis of adult human heart tissue with a particular focus on frozen tissue. We were also interested in establishing a method for downsampling of cardiomyocytes to allow targeted studies of other cell types, which may be more important for damage responses and remodelling of the heart as well as mediation of a range of diseases.

## 2 Materials and methods

### 2.1 Tissue collection and handling

To validate the effectiveness of protocols for single-cell isolation in mouse hearts, hearts were obtained from C57BL/6 mice sedated with isoflurane and were immediately placed on ice in cold PBS, perfused with PBS and dissected. Samples were then processed before single cell isolation with three different protocols: processed fresh, snap-frozen, or treated with an RNA-stabilizing agent (RNAlater, ThermoFisher Scientific, Waltham, MA, USA) and subsequently frozen. Frozen samples were kept at -80 °C and only thawed at the time of cell isolation. All animal procedures were approved by the local ethics committee in Malmö and Lund for animal research.

For human heart tissue, biopsies from the left ventricular free wall were obtained from the explanted heart of four patients undergoing orthotopic heart transplantation, of which three with dilated cardiomyopathy (DCM) and one with ischemic cardiomyopathy. Sectioning from areas with grossly discernible scar tissue was avoided. Biopsies were sectioned, immediately placed in RNA-later, frozen to -80 °C, stored for approximately 12 months, and only thawed for cell isolation. Written informed consent was obtained from all individuals and the local ethics committee approved of the study. For RNA-sequencing of single nuclei, two separate biopsies from one patient with DCM were used.

To compare the expression profiles of cellular, cytoplasmic and nuclear RNA profiles, we used commercially available primary human cardiac fibroblasts (hcFB, Cell Applications Inc., San Diego, USA), human cardiac microvascular endothelial cells (hcMVEC, Lonza), and human cardiomyocytes derived from induced pluripotent stem cells (iPS-CM, Cellular Dynamics International, Madison, WI, USA). Cells were cultured in HCF Growth Medium (Cell Applications Inc), Endothelial basal medium (Lonza) and iCell Cardiomyocyte Plating or Maintenance Medium (Cellular Dynamics International), respectively. HcMVEC cells were plated at a density of 30,000 cell per well in 6 well plates while hcFB and iPS-CM were plated at a density of 200,000 cells per well in 6 well plates.

### 2.2 Enzymatic tissue digestion

Five different protocols for enzymatic digestion and cell isolation of cardiac tissue was assessed in murine and human heart tissue, based on previous protocols applied to mouse tissue (Farbehi et al., 2019; Gladka et al., 2018) and one which had been used on human tissue (Bajpai et al., 2018). Dissected tissue was placed in a tube and incubated with one of the following: (1) HBSS with 1 mg/ml collagenase type II (ThermoFisher Scientific, Waltham, MA, USA) and 3 mM CaCl_2_, (2) HBSS with 0.5 mg/ml Elastase (Sigma Aldrich, St Louis, MO, USA) and 3 mM CaCl_2_, (3) HBSS with 1 mg/ml Collagenase Type II and 0.5 mg/ml Elastase, (4) DMEM with 450 U/ml Collagenase Type I and 60 U/ml Hyaluronidase or (5) DMEM with Liberase (Roche, Basel, Switzerland). Tissue was digested for 2 h at 37 °C in protocol 1–3 and 1 h at 37 °C in protocol 4–5. Mouse tissue was digested for 20 min in all protocols. After incubation, cells were passed through 100 or 200 μM cell strainers, washed with HBSS and resuspended in medium for primary cells.

### 2.3 Flow cytometry and high content screening

After enzymatic tissue digestion, cells were collected by centrifugation at 300g for 10 min at room temperature. Cells were fixed in 4% phosphate buffered formaldehyde for 10 min at 37 °C followed by incubation on ice for 1 min, permeabilization by addition of 1% saponin, incubation for 30 min on ice, and washing with 2 ml incubation buffer (PBS with 0.5% BSA). Cells were re-suspended in incubation buffer and incubated with antibodies for PCM1 (1:500, Sigma Aldrich, St Louis, MO, USA) for 1 h. After two washes cells were stained with secondary antibodies, anti-mouse Alexa 555 (1:1000, Life technologies, Carlsbad, CA, USA), anti-rabbit Alexa 488 (1:1000, Cell Signalling Technology, Danvers, MA, USA) and Vimentin Alexa 647 antibody (1:100, Cell Signalling Technology, Danvers, MA, USA). Cells were analysed with a flow cytometry sorter (BD FACSAria III, BD Biosciences, Franklin Lakes, NJ, USA).

For high content screening microscopy, single PCM1^+^ and PCM1^-^ nuclei as well as cells isolated from different enzyme digestion protocols were stained with DRAQ5 DNA stain at 10 μM final concentration and analyzed morphologically and quantitatively through automated image analysis based on DRAQ5 staining using Cellomics ArrayScan VTI HCS Reader (ThermoFisher Scientific, Waltham, MA, USA) at 10x and 20x respectively. Counts were averaged across 50 individual frames per sample and manually curated.

### 2.4 Nuclear isolation, flow cytometry and sorting

Nuclear isolation was performed as previously described (Bergmann and Jovinge, 2012) Briefly, approximately 50 mg of cardiac tissue was dissected with a scalpel, suspended in lysis buffer (0.32 M Sucrose, 10 mM Tris-HCL (pH 8), 5 mM CaCl_2_ 5 mM Magnesium Acetate, 2 mM EDTA, 0.5 mM EGTA, 1mM DTT) and homogenized first using a Omni TH rotor-stator followed by eight strokes with a Dounce homogenizer. Tissue homogenate was then passed through 100 and 70 μm strainers (BD Biosciences), centrifuged at 700g for 10 min at 4 °C and resuspended in sucrose buffer (2.1 M Sucrose, 10 mM Tris-HCl (pH 8), 5 mM Magnesium Acetate, 1 mM DTT). The nuclear suspension was carefully layered on top of fresh sucrose buffer and centrifuged at 13,000g for 60 min at 4 °C. The pellet was dissolved in nuclei storage buffer (0.44 M Sucrose, 10 mM Tris-HCl (pH 7.2), 70 mM KCl, 10 mM MgCl_2_, 1.5 mM Spermine).

Isolated nuclei were stained with a PCM1 antibody (1:500, Sigma, HPA) for 1 h at 4 °C. Cells were washed twice with nuclei storage buffer and stained with a secondary antibody Alexa Fluor 488 anti rabbit (1:1000, Cell signaling) for 1 h at 4 °C and then stained with Draq5 (1:300, BioStatus, Shepshed, UK).

Nuclei were analysed by the flow cytometry sorter BD FACSAria III and were gated based on forward scatter, Draq5 and PCM1.

### 2.5 RNA isolation, array-based and qPCR-based expression analysis of isolated nuclei

Total RNA was isolated from human cardiac tissue, cells and sorted nuclei with the miRNeasy mini Kit (Qiagen, Hilden, Germany) according to the manufacturer's instructions. Tissue was homogenized in QIAzol using an Omni TH rotor-stator homogenizer before RNA isolation. Nuclear and cytoplasmic RNA from cultured cells was prepared using the Cytoplasmic & Nuclear RNA Purification Kit according to the manufacturer's instructions (Norgen, Ontario, Canada). RNA quantity was determined with NanoDrop (Agilent, Santa Clara, CA, USA). RNA integrity was assessed using the Agilent RNA 6000 Pico assay on a Bioanalyzer 2100 (Agilent). RNA integrity numbers ranged from 6-9 (mean RIN: 6.5).

2 ng of total RNA from sorted PCM1^+^ and PCM1^-^ nuclei was reverse transcribed with random hexamer primers using the Revert Aid H Minus First Strand cDNA Synthesis Kit (ThermoFisher Scientific). Quantitative real-time PCR was performed using TaqMan assays for cardiac Troponin I (*TNNI3*, Hs00165957), Pericentriolar Material 1 (*PCM1*, Hs00196390), Vimentin (*VIM*, Hs00958111) or Glyceraldehyde-3-Phosphate Dehydrogenase (*GAPDH*, Hs02786624) and TaqMan Universal Master Mix II on a StepOnePlus instrument (ThermoFisher).

Total, cytoplasmic and nuclear RNA from iPS-CM, hcFB, hcMVEC and total RNA from PCM1^+^ and PCM1^-^ sorted nuclei was analyzed in triplicates using the Clariom D transcriptome profiling microarray (Affymetrix Inc, Santa Clara, CA, USA). cRNA synthesis and hybridization was performed using the GeneChip WT Pico Kit with 10 ng of RNA/sample as input and following the manufacturers recommendations. Probe summarization and data normalization was performed using the Robust Multi-array Analysis (RMA) and signals were log 2 transformed. An expression threshold was defined based on the signal of negative control probes included on the array targeting intronic sequences. Any transcript with a signal above the 95^th^ percentile of the averaged negative control probe signal was considered to be expressed. All transcripts annotated as “Coding” were selected for the analysis. Two samples (one PCM1^+^ nuclear sample and one total RNA sample from hcMVEC) were excluded from the analysis based on aberrant transcriptome profiles.

### 2.6 Library preparation and single-cell RNA sequencing

Nuclear suspensions were loaded into the 10x Genomics Chromium Controller microdroplet-based microfluidic instrument (10x Genomics Inc, San Francisco, CA) at the Eucaryotic Single Cell Genomics facility of SciLife Labs (Stockholm, Sweden). Briefly, this system performs encapsulation of single cells or nuclei into barcoded microdroplets using a Gel bead in EMulsion (GEM) (Zheng et al., 2017). Each gel bead is also labeled with oligonucleotides consisting of a unique 14-basepair barcode, a 10-basepair unique molecular identifier (UMI), adapters and primers for sequencing, and an anchored 30-basepair oligo-dT to prime polyadenylated RNA transcripts. Reverse transcription then takes place in each droplet, after which barcoded complementary DNAs (cDNAs) are combined and amplified for library preparation in bulk. Libraries were prepared for an estimated 4000 nuclei per sample according to instructions from the manufacturer. Sequencing of libraries was then conducted on a NovaSeq 6000 sequencing system (Illumina Inc, San Diego, CA) targeting >50 000 reads per cell.

Raw base calls from the sequencing system were obtained as Illumina .bcl files and read into the Cellranger software v2.1.1 (10x Genomics, Pleasanton, CA, USA) for demultiplexing, mapping, and quality control. Reads were aligned to the human reference genome build GRCh38, based on a custom-made gene annotation file including pre-mRNAs. Barcodes with no reads were filtered out. The resulting gene count matrices were reviewed and nuclei with over 300 and under 2000 features were retained for further analysis.

### 2.7 Normalization, dimensionality reduction and cluster analysis

The gene count matrices were read into Seurat v 3.0 (Stuart et al., 2019) for analysis, creating objects based upon cells containing at least one feature and features present in at least one cell. For the first biopsy, a total of 28,441 features were included across 8,460 nuclei. For the second PCM- filtered biopsy, a total of 22,030 features were included across 4,760 nuclei. In Seurat, molecular counts were normalized for differences in sequencing depth across nuclei with variance stabilization using regularized negative binomial regression as implemented in the sctransform algorithm (Hafemeister and Satija, 2019). Dimensionality reduction was then conducted through principal component analysis. Based on the first 50 principal components, two-dimensional projections were created using the UMAP (Uniform Manifold Approximation and Projection for dimension reduction) technique (Becht et al., 2018). Graph-based clustering of nuclei was conducted based on a shared nearest neighbor graph of the UMAP projection followed by modularity optimization using the smart local moving (SLM) algorithm described by Waltman and van Eck (Waltman and Van Eck, 2013) with a resolution parameter of 0.1. The impact on projected clusters of tuning the two primary UMAP parameters (numbers of neighbors and minimum distance) was evaluated. Expression of conventional cell-type informative markers typically used in immunohistochemistry experiments were explored across clusters and visualized quantitatively in dotplots and UMAP graphs.

### 2.8 Statistical analysis

For comparison of nuclear with whole-cell and cytoplasmic transcriptomes as well as transcriptomes from bulk tissue, Pearson's correlation coefficient and Lin's concordance correlation coefficient was calculated across different expression thresholds and plotted. Bland-Altman plots with limits of agreement were also derived.

To study the efficiency of PCM1-gating, PCM1+ and PCM1- cells were compared for *TNNT3* and *VIM* expression using the Mann-Whitney U test.

Enrichment of gene ontology project pathways (Ashburner et al., 2000) within each cluster of nuclei as compared to other nuclei was examined, based on genes with at least a log fold difference of 0.7 between clusters (n = 1,042), using hypergeometric tests as implemented in the R package topGO (v2.38.1).

All datasets are available in full through the Gene Expression Omnibus (GEO) database at the National Center for Biotechnology Information (NCBI, http://www.ncbi.nlm.nih.gov/geo, accession code GSE161067 for the microarray dataset and GSE161153 for the single nucleus RNA-sequence dataset).

## 3 Results

### 3.1 Evaluation of protocols for enzymatic digestion of heart tissue

We first evaluated five different protocols for enzymatic digestion and single cell isolation of heart tissue, based on previous protocols applied to mouse tissue (Farbehi et al., 2019; Gladka et al., 2018) and one which had been applied to human tissue, although without specifying the quality of the resulting cell suspension (Bajpai et al., 2018). Approximately 50 mg of left ventricular tissue was extracted from healthy mice and explanted human hearts and subjected to enzymatic digestion and cell isolation. High quality single cell data from enzymatically dissociated tissue relies on achieving a cell suspension with a minimum of clumps, dead or dying cells and extracellular debris (Haque et al., 2017). The quality of the single cell suspensions was therefore analyzed quantitatively and morphologically using DRAQ5 staining followed by flow cytometry and high content screening (HCS) digital fluorescence microscopy.

**Figure 1:**
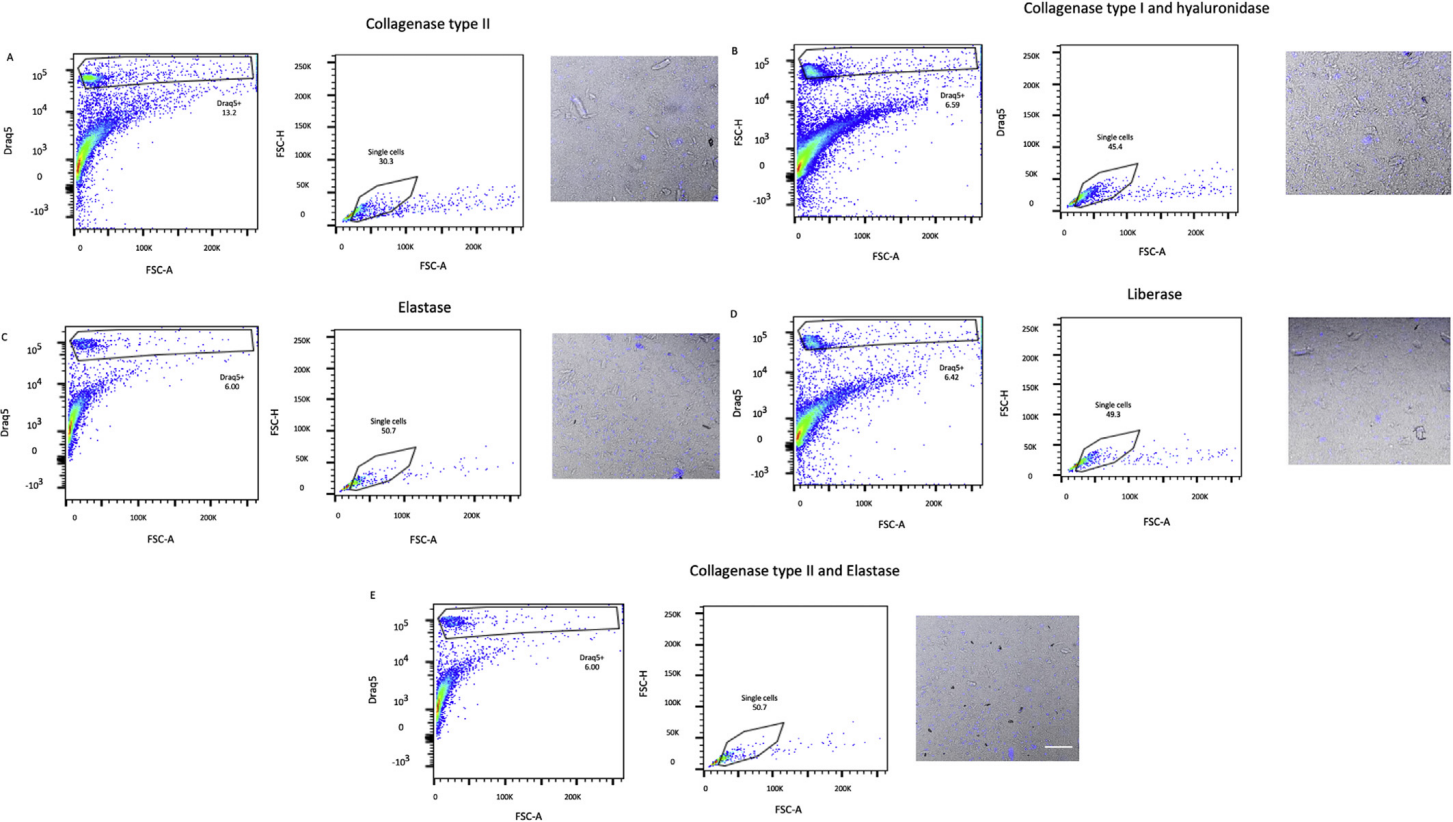
Flow cytometry analysis of mouse cells isolated from frozen heart tissue samples pretreated with an RNA-stabilizing agent. Murine heart tissue was digested with (A) collagenase type II, (B) collagenase type I and hyaluronidase, (C) elastase, (D) Liberase, or (E) collagenase type II and elastase, and analysed by flow cytometry and high content screening (HCS). Isolated cells were identified and gated first by forward scatter area (FSC-A) vs DRAQ5, second on FSC-A vs FSC-height (FSC-H) to distinguish single cells. The morphology and integrity of DRAQ5-stained cell suspensions were analysed using a HCS fluorescent microscope (scale bar = 200 μm). Prior to digestion, hearts had been treated with an RNA-stabilizing agent and frozen to -80 °C.

**Figure 2:**
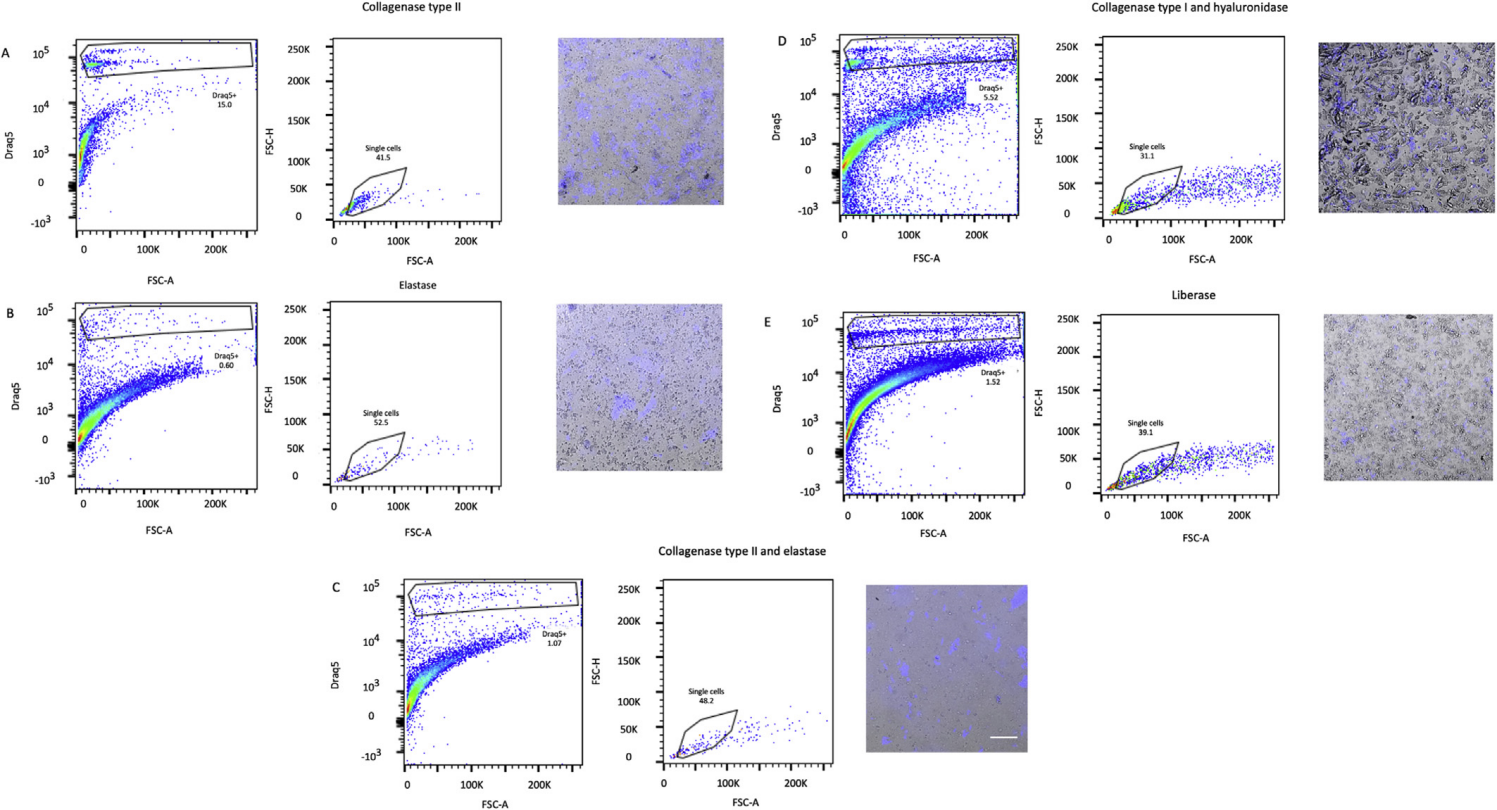
Flow cytometry analysis of mouse cells isolated from snap frozen heart tissue samples. Murine heart tissue was digested with (A) collagenase type II, (B) collagenase type I and hyaluronidase, (C) elastase, (D) Liberase, or (E) collagenase type II and elastase, and analysed by flow cytometry and high content screening (HCS). Isolated cells were identified and gated first by forward scatter area (FSC-A) vs DRAQ5, second on FSC-A vs FSC-height (FSC-H) to distinguish single cells. The morphology and integrity of DRAQ5-stained cell suspensions were analysed using a HCS fluorescent microscope (scale bar = 200 μm).

**Figure 3:**
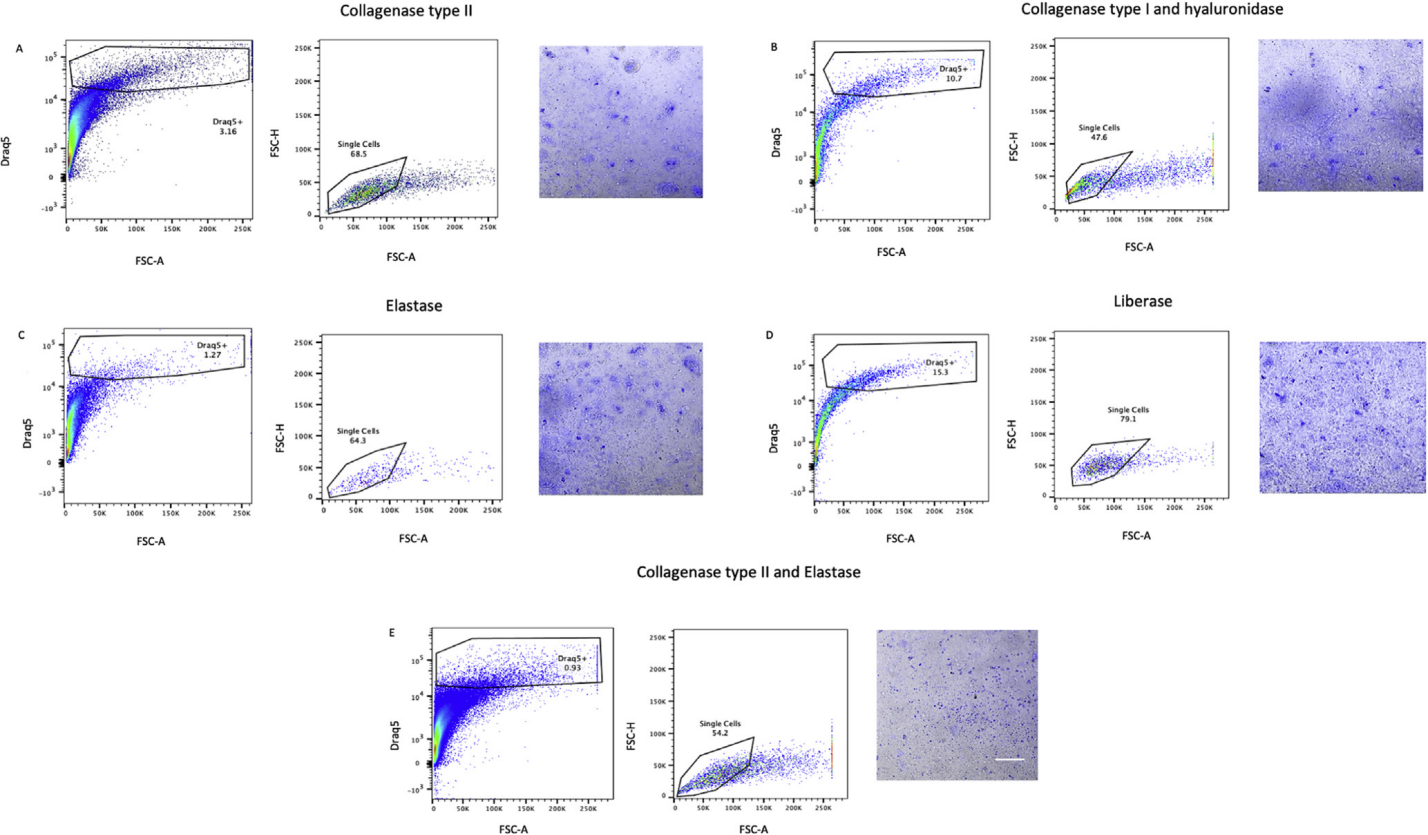
Flow cytometry analysis of human cells isolated from frozen heart tissue samples pretreated with an RNA-stabilizing agent. Human heart tissue was digested with (A) collagenase type II, (B) collagenase type I and hyaluronidase, (C) elastase, (D) Liberase, or (E) collagenase type II and elastase, and analysed by flow cytometry and high content screening (HCS). Isolated cells were identified and gated first by forward scatter area (FSC-A) vs DRAQ5, second on FSC-A vs FSC-height (FSC-H) to distinguish single cells. The morphology and integrity of DRAQ5-stained cell suspensions were analysed using a HCS fluorescent microscope (scale bar = 200 μm). Prior to digestion, hearts had been treated with an RNA-stabilizing agent and frozen to -80 °C.

**Figure 4:**
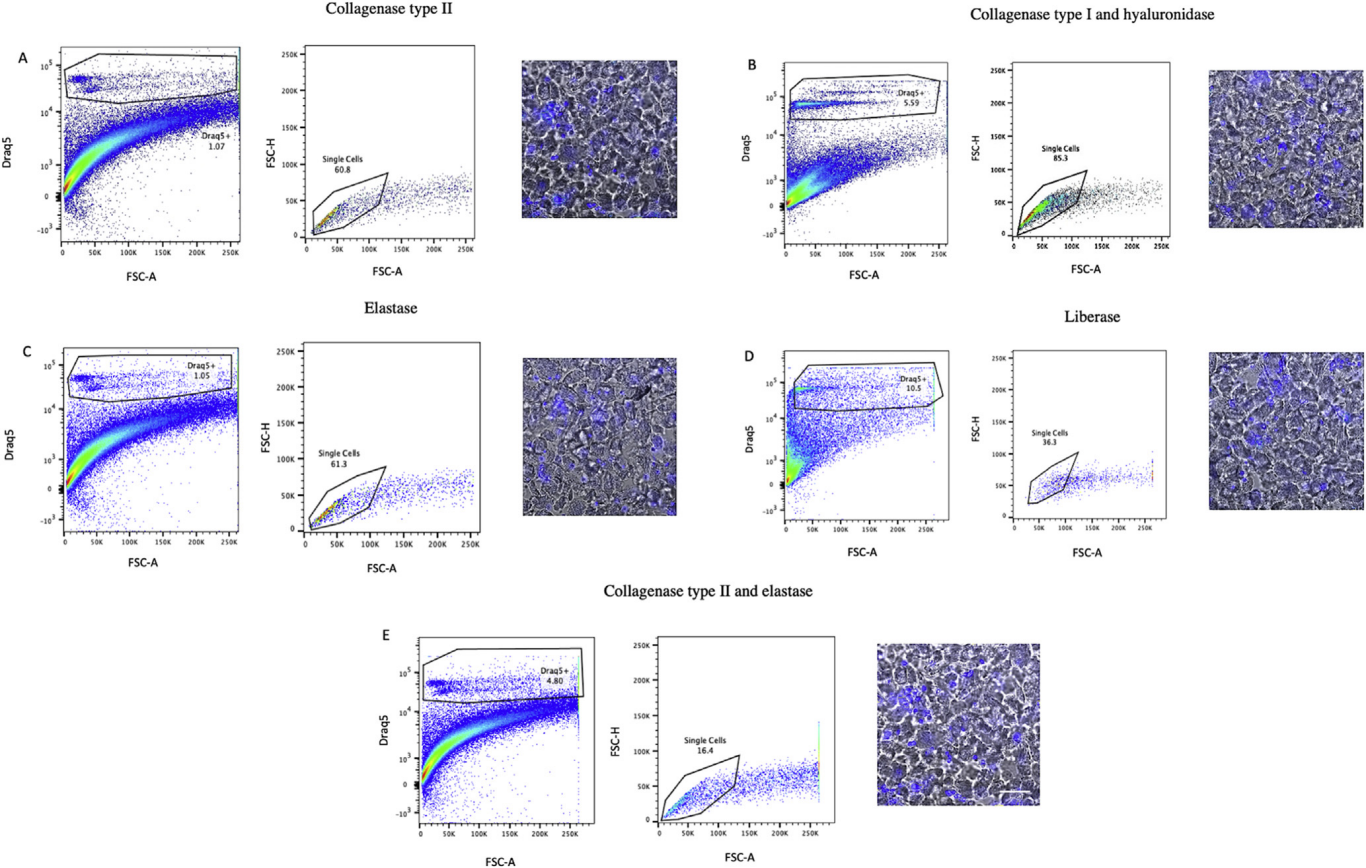
Flow cytometry analysis of mouse cells isolated from fresh heart tissue. Murine heart tissue was digested with (A) collagenase type II, (B) collagenase type II and hyaluronidase, (C) elastase, (D) Liberase, or (E) collagenase type II and elastase, and analysed by flow cytometry and high content screening (HCS). Isolated cells were identified and gated first by forward scatter area (FSC-A) vs DRAQ5, second on FSC-A vs FSC-height (FSC-H) to distinguish single cells. The morphology and integrity of DRAQ5-stained cell suspensions were analysed using a HCS fluorescent microscope (scale bar = 200 μm).

For each of the protocols, digestion of frozen mouse hearts regardless of whether snapfrozen or treated with RNA-stabilizing agent did not result in distinct DRAQ5^+^ populations when analyzed with flow cytometry (Figures 1 and 2) and HCS identified 0–4 intact cells/ul of cell suspension (Table 1). Similarly, even though digestion of frozen human tissue resulted in a number of DRAQ5^+^ events, HCS revealed that the suspensions were absent of intact cells and displayed a high degree of debris (Figure 3). In contrast, digestion of fresh mouse hearts yielded higher numbers of cells, especially with the Liberase-based protocol (Figure 4). Based on these results, we conclude that standard means of enzymatic dissociation is not sufficient to produce single cell suspensions from human and murine hearts when the tissue has been frozen. As freezing will be required for analysis of human hearts at scale, alternative methods will be required.

**Table 1:** Count of isolated cells from human and mouse hearts with different protocols for enzymatic digestion and tissue handling.

Enzyme digestion protocols	Valid cell objects (no/uL)			
	*Human, RNAlater*	*Mouse, RNAlater*	*Mouse, snapfrozen*	*Mouse, fresh*
Collagenase type II	1.3	2	2.2	7.2
Elastase	0	0	0	2.5
Collagenase type II + Elastase	0	2	1	2.7
Collagenase type I + Hyaluronidase	0	3	4	6.1
Liberase	0.2	2	3	13.2

Valid cell objects counted automatically and curated manually based on DRAQ5 DNA staining. Cells were counted automated image analysis in a high-throughput screening microscope.

### 3.2 Single nuclei isolation

**Figure 5:**
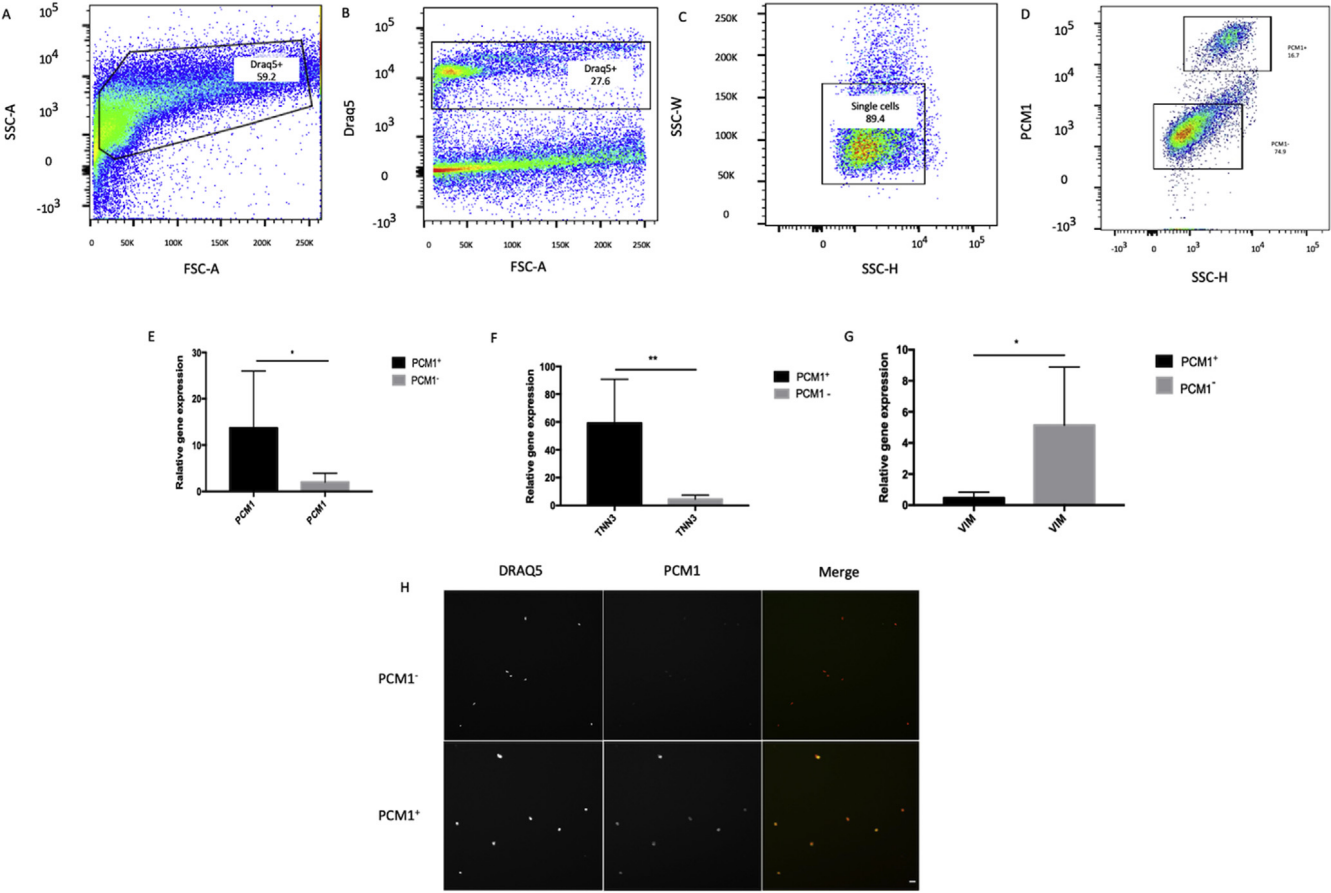
Evaluation of nuclei isolated from human cardiac left ventricle tissue. (A) Nuclei were first identified by forward scatter area (FSC-A) vs side scatter area (SSC-A). (B) In the second gate DRAQ5 positive nuclei were separated from debris. (C) Single nuclei were gated by SSC-height (SSC-H) versus SSC-width (SSC-W). (D) Cardiomyocyte nuclei were separated from non-cardiomyocyte nuclei based on PCM1-staining. (E,F,G) Gene expression of sorted nuclei revealed that the PCM1^+^ fraction was enriched for the cardiomyocyte marker genes *PCM1* and *TNNT3*, whereas the cardiac fibroblast marker gene *VIM* was enriched in the PCM1^-^ fraction. Data is presented as mean ± SD from three independent experiments. (H) FACS-sorted nuclei, stained with DRAQ5 and PCM1 were scanned with high content screening microscopy. Scale bar = 10 μm.

Transcriptomic analysis of single nuclei has been reported as a viable alternative to single cell analysis for tissues that cannot be readily dissociated (Habib et al., 2016; Lacar et al., 2016; Lake et al., 2016). To test this strategy in the context of heart tissue, we evaluated a previously described protocol for isolation of single nuclei from human cardiac tissue, based on sucrose-induced cell lysis, density gradient centrifugation and a series of homogenization steps (Bergmann and Jovinge, 2012). We applied this protocol to left ventricular biopsies from explanted hearts (n = 3) and assessed the quality and purity of the nuclear suspension with flow cytometry and HCS. We observed a distinct population of DRAQ5^+^ nuclei with flow cytometry with an average yield of 40,000 nuclei/gram of tissue. HCS showed that the nuclear suspension was virtually free of debris and aggregates (Figure 5).

To improve the sensitivity in the analysis of non-cardiomyocytes and further reduce the risk of including debris, we employed a strategy of sorting dissociated nuclei based on the cardiomyocyte marker PCM1 as previously suggested (Bergmann and Jovinge, 2012; Bergmann et al., 2011; Preissl et al., 2015, See et al., 2017). Distinct DRAQ5+/PCM1+ and DRAQ5+/PCM1- nuclear populations were observed with flow cytometry (Figure 5). The purity and cellular origin of sorted PCM1+ and PCM1- nuclei was then assessed by qPCR. Gene expression analysis of canonical cardiomyocyte and fibroblast markers (Troponin T, *TNNT3* and Vimentin, *VIM*, respectively) on sorted nuclei revealed that PCM1^+^ nuclei displayed a 60-fold enrichment in *TNNT3* whereas PCM1- nuclei showed a 6-fold enrichment in *VIM* expression, confirming the cardiomyocyte origin of the former (Figure 5).

**Figure 6:**
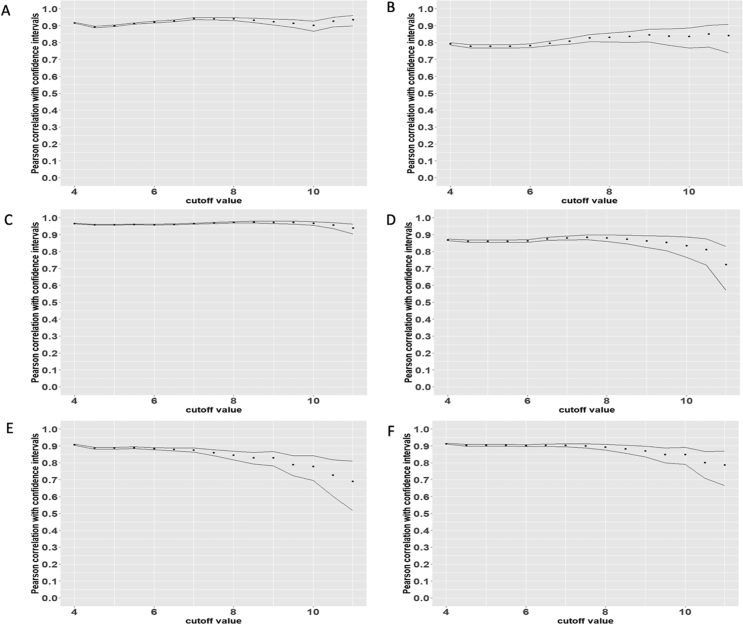
Correlation of nuclear with cytoplasmic and whole-cell transcriptomes. Pearson's correlation coefficients with 95% confidence intervals for nuclear transcriptomes with cytoplasmic and whole-cell transcriptomes in induced pluripotent stem cell derived cardiomyocytes (cytoplasmic, A; whole-cell, B), primary human cardiac fibroblasts (cytoplasmic, C; whole-cell, D), and cardiac microvascular endothelial cells (cytoplasmic, E; whole-cell, F). X-axis indicates different thresholds for non-expressed transcripts representing technical noise.

Next, we sought to validate that the RNA content of nuclei faithfully mirrored that of whole tissue and that of individual cardiac cells. To this end, we performed array-based transcriptomic profiling of RNA from bulk heart biopsies, sorted PCM1^+^ and PCM1^-^ nuclei, as well as cytoplasmic, nuclear and total RNA fractions from three human cardiac cell types: induced pluripotent stem cell-derived cardiomyocytes (iPS-CMCs), primary cardiac fibroblasts and primary cardiac microvascular endothelial cells (n = 3) by whole transcriptome microarray. Array-based profiling, although an older method, provides high reproducibility and results that are generally highly correlated with RNA-sequencing but with the additional advantage of lower sensitivity to differences in high- and low-abundance transcripts between samples which may be particularly relevant when comparing cytoplasm with nuclei (Nazarov et al., 2017). We found that nuclear transcriptomes closely correlated with cytoplasmic and whole-cell transcriptomes for both iPS-CMCs, primary fibroblasts and primary endothelial cells (Figure 6). Varying the lower expression threshold had limited impact on point estimates for correlation coefficients although a higher threshold resulted in wider confidence intervals, especially in endothelial cells. We also observed high absolute concordance of nuclear transcriptomes with cytoplasmic and whole-cell transcriptomes (Figure S1). Furthermore, the PCM1^+^ nuclear transcriptome was more strongly correlated with the bulk tissue transcriptome than was the PCM1^-^ nuclear transcriptome (Pearson's r = 0.8 and 0.65, respectively, Figure S2), consistent with the notion of predominant cardiomyocyte representation in the bulk cardiac transcriptome. As expected, iPS-CMCs were also more strongly correlated with PCM1+ than PCM1- (r = 0.8 and 0.65) supporting the validity of these cells as proxy for cardiomyocytes (Figure S2).

### 3.3 Single nuclei RNA-sequencing of a human heart

We next applied our protocol to generate a single-cell transcriptional profile of a human heart.

**Figure 7:**
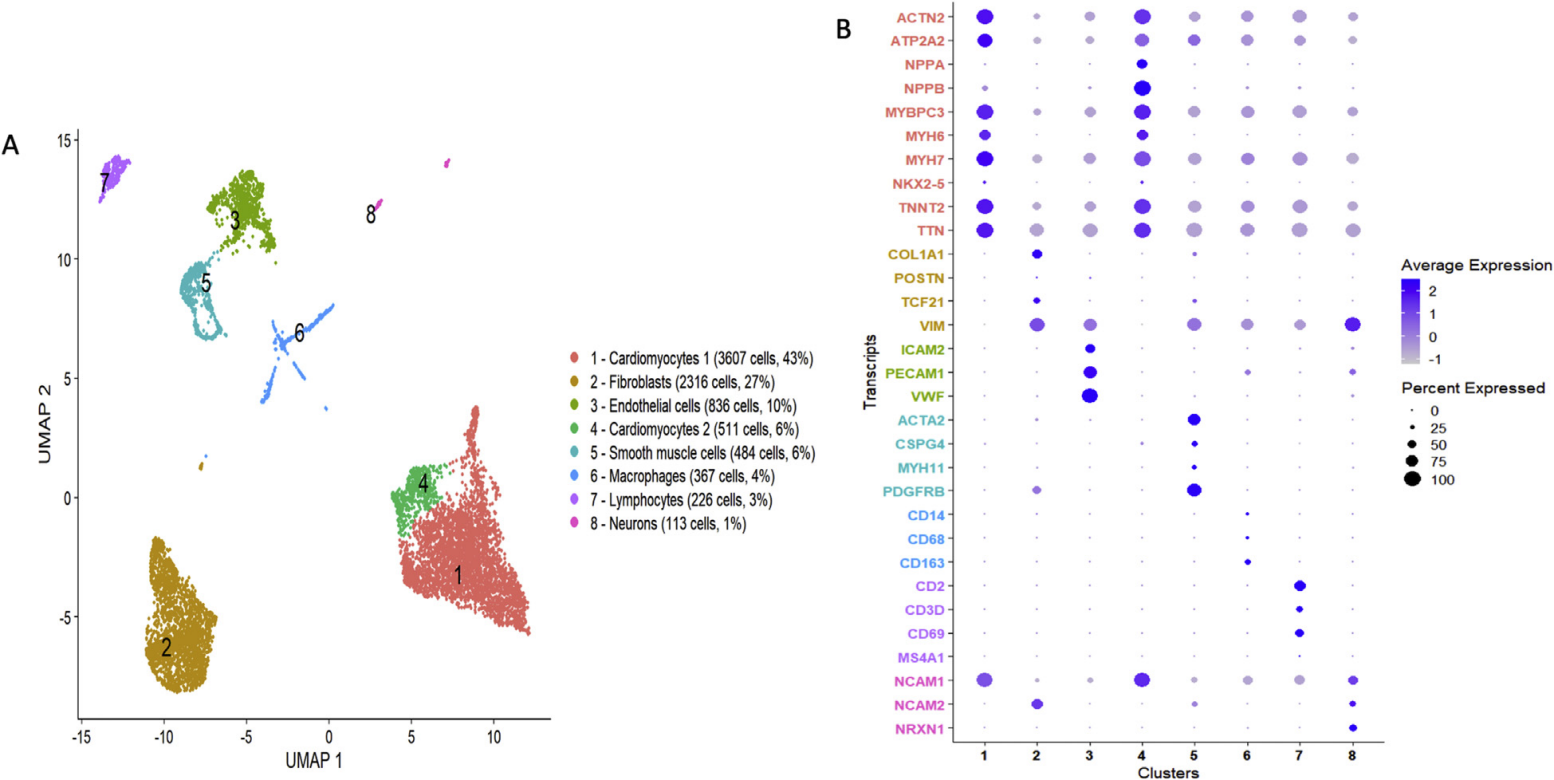
RNA-sequencing of human heart cells. Panel A shows a Uniform Manifold Approximation and Projection (UMAP) plot based on RNA-sequencing of 8,460 individual nuclei from a human heart. Of the nuclei, the proportion of cardiomyocyte nuclei has been enriched such that 4,390 had been selected from PCM1^+^ and 4,070 from PCM1^-^ nuclear fractions by fluorescence-activated cell sorting. Panel B shows a dot plot of expression across clusters of preselected, established marker genes representing the major expected cell types in human heart: cardiomyocytes (red), fibroblasts (yellow), endothelial cells (green), vascular smooth muscle cells and pericytes (cyan), macrophages (blue), lymphocytes (purple), and neurons (pink).

**Figure 8:**
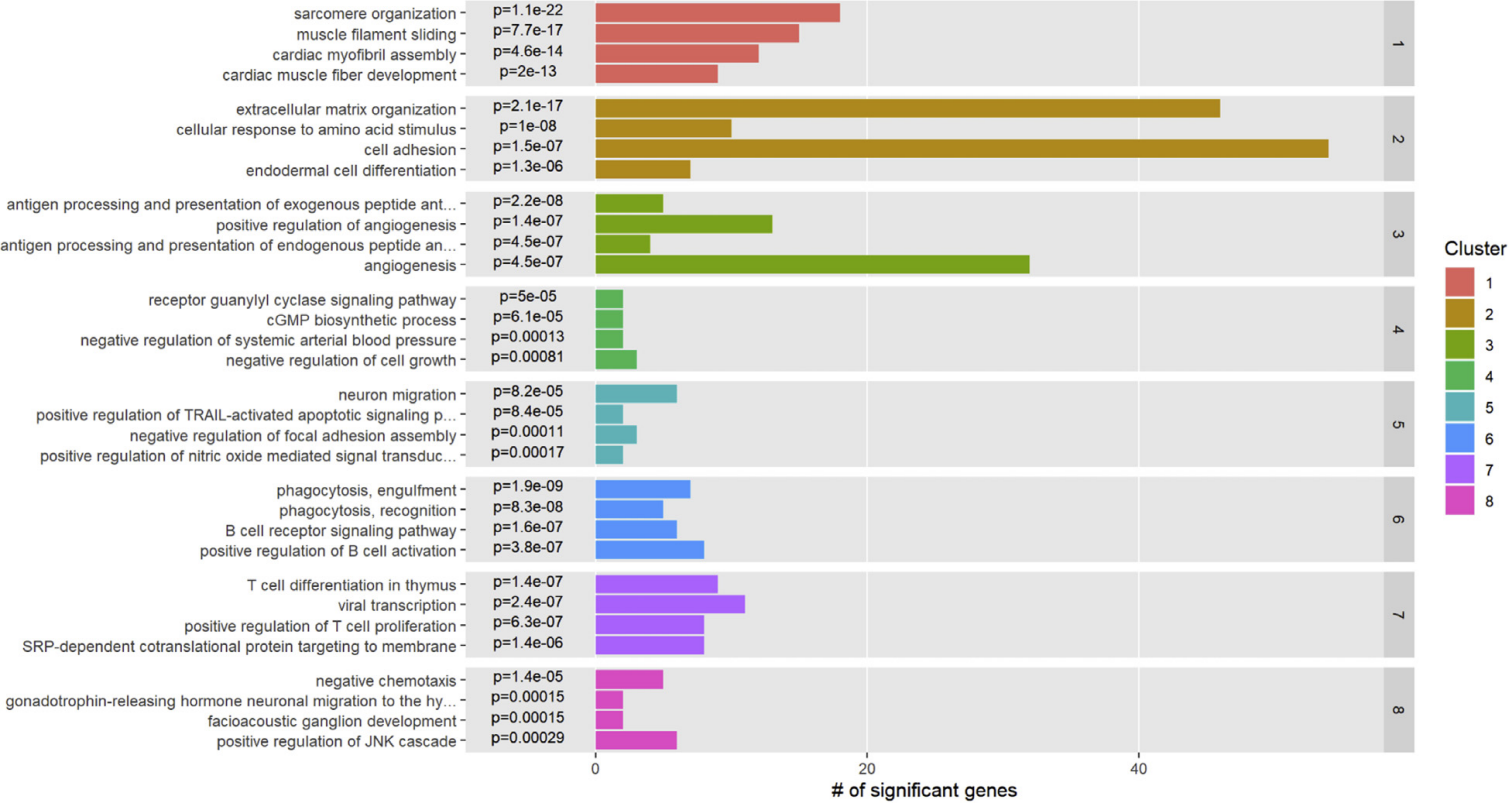
Pathway enrichment analysis of single-nucleus clusters. Pathway enrichment analysis across clusters, incorporating all expressed transcripts and pathway annotations from the Gene Ontology project.

An explanted heart from a patient with dilated cardiomyopathy (DCM) was used to isolate nuclei following the protocol outlined above. Structural integrity of nuclei and absence of debris and nuclear aggregates was confirmed by brightfield microscopy with Hoechst staining (Figure S3) and flow cytometry. In total, 29% of nuclei displayed PCM1+ staining in FACS indicating cardiomyocyte identity, similar to other studies (Molenaar and van Rooij, 2018). Transcriptional profiling of single nuclei with RNA sequencing (sncRNAseq) was performed in 4,390 PCM1^+^ and 4,070 PCM1^-^ nuclei in aggregate, using a commercially available microdroplet-based microfluidic platform (Chromium Controller, 10x Genomics, San Francisco, CA). A total of 457,059,241 reads was generated, with a mean of 54,026 reads and a median of 2608 genes per nucleus. In cluster analyses based on Uniform Manifold Approximation and Projection (UMAP) projections of the combined PCM1+ and PCM1- nuclei, we identified eight distinct clusters (Figure 7A) that were consistently observed across different UMAP parameters (Figure S4). Conventional marker genes typically used in immunohistochemistry (Figure 7B) and Gene Ontology pathway analyses (Figure 8) indicated cluster identities consistent with established cardiac cell types: cardiomyocytes (clusters 1 and 4), fibroblasts (cluster 2), endothelial cells (cluster 3), vascular smooth muscle cells (VSMCs, cluster 5), macrophages (cluster 6), lymphocytes (cluster 7) and neurons (cluster 8). Of particular note, we observed clustering of cardiomyocytes into two separate clusters, the smaller of which was defined specifically by high natriuretic peptide expression. In the fibroblast cluster, *TCF21* was widely expressed, previously shown to represent tissue-resident fibroblasts (Kanisicak et al., 2016), while a smaller, relatively distinct subcluster displayed *POSTN* expression indicating an activated myofibroblast phenotype. Similarly, for macrophages, only a few cells expressed CCR2 which has been suggested to be a marker for non-tissue resident macrophages recruited from the circulation (Bajpai et al., 2018). Expression profiles of marker genes (Figure 7B) further suggested clustering of pericytes (*PDGFRB*, *CSPG4*) within the VSMC cluster, NK cells (*FCGR3A* [CD57]) and adipocytes (*ADIPOQ* [adiponectin]) in the macrophage cluster, and the presence of both B (*MS4A1* [CD20]) and T cells (*CD3D*, *CD69*) in the lymphocyte cluster. Of these cell types, only adipocytes formed a relatively distinct subcluster (Figure S5).

**Figure 9:**
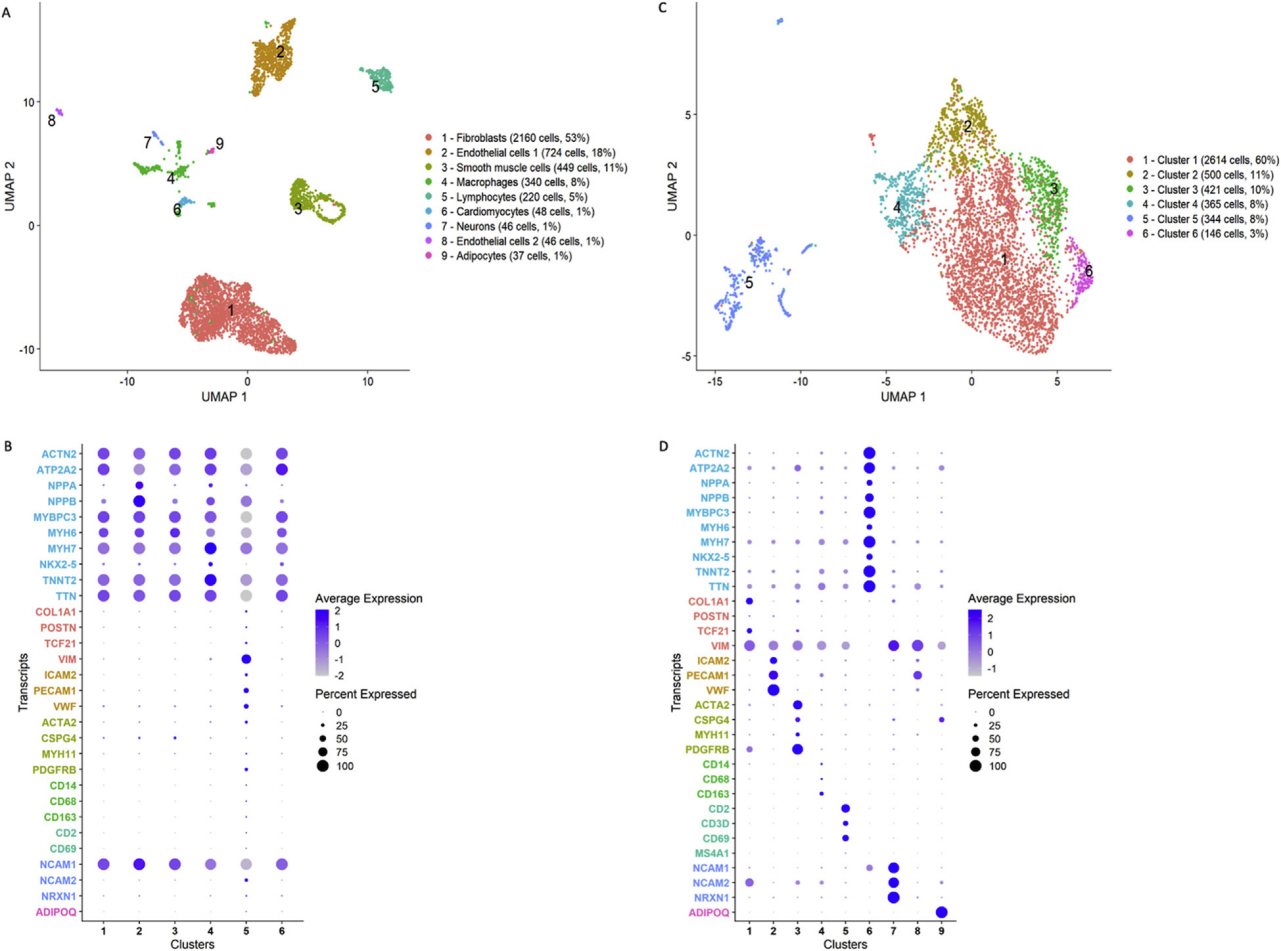
Clustering of nuclei by PCM1-staining. Uniform Manifold Approximation and Projection (UMAP) projections based on RNA-sequencing of 4,070 PCM1^-^ (panels A and B) and 4,390 PCM1^+^ (C and D) nuclear fractions from fluorescence-activated cell sorting. Below UMAP plots are corresponding dot plots (panels B and D) illustrating expression across clusters of preselected, established marker genes representing the major expected cell types in human heart, with color codes representing the PCM1^-^ clusters: cardiomyocytes (cyan), fibroblasts (red), endothelial cells (yellow), vascular smooth muscle cells and pericytes (light green), leukocyte populations (dark green), neurons (blue) and adipocytes (pink).

We next stratified sequenced cells based on nuclear PCM1 staining, to evaluate the validity of this method for downsampling of cardiomyocytes (Figure 9). In the cardiomyocyte-enriched (PCM1^+^) sample one predominant cluster was observed, including 92% of all nuclei, which corresponded to cardiomyocytes in marker gene analysis, while a small non-cardiomyocyte cluster included a mix of most cell types. In the cardiomyocyte-depleted (PCM1^-^) sample only a small cluster of cardiomyocytes was observed, representing 1% of nuclei, while fibroblasts represented the majority of nuclei (53%), followed by endothelial cells (19%) and smooth muscle cells (11%). Furthermore, we observed clear separation of two clusters representing endothelial cells and a cluster representing adipocytes. We noted considerably higher sequencing saturation (82%) in the cardiomyocyte-depleted as compared to the cardiomyocyte-enriched (50%) sample, consistent with higher RNA content in cardiomyocytes, dominated by high abundance of sarcomeric and mitochondrial transcripts.

**Figure 10:**
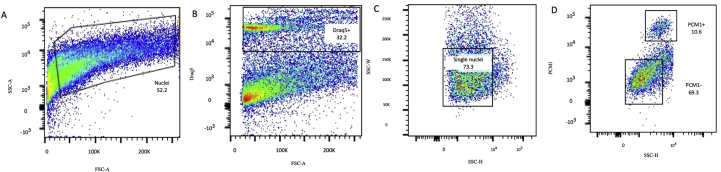
Flow cytometry analysis of a second sample from the same human heart. (A) Nuclei were first identified by forward scatter area (FSC-A) vs side scatter area (SSC-A). (B) In the second gate DRAQ5 positive nuclei were separated from debris. (C) Single nuclei were gated by SSC-height (SSC-H) versus SSC-width (SSC-W). (D) Cardiomyocyte nuclei were separated from non-cardiomyocyte nuclei based on PCM1-staining.

**Figure 11:**
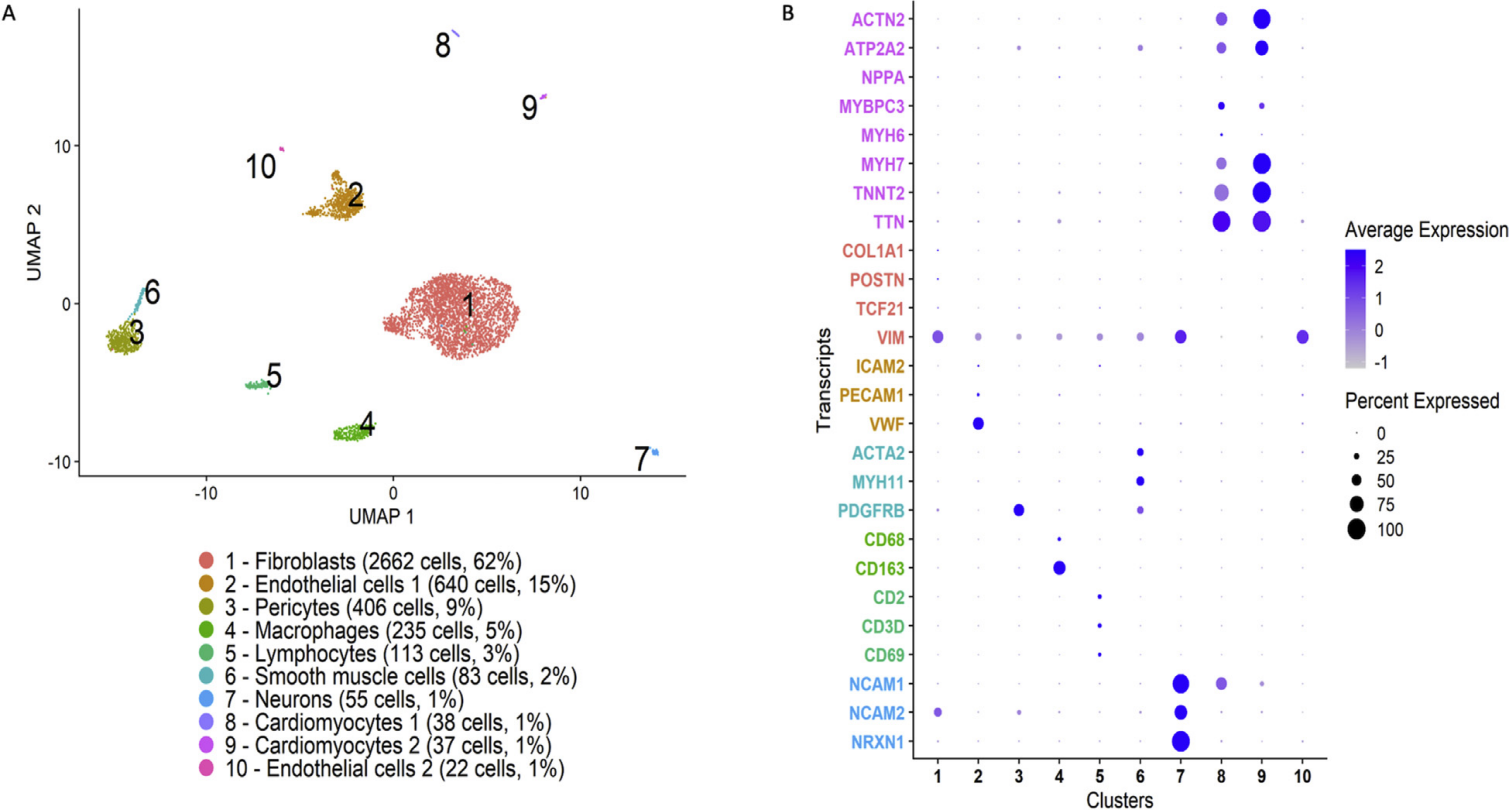
Clustering of nuclei from the second sample. Uniform Manifold Approximation and Projection (UMAP) projection based on RNA-sequencing of 4,760 PCM1^-^ nuclei from fluorescence-activated cell sorting of a second, separate biopsy from the same heart as in Figure 4. The corresponding dot plot illustrates expression across clusters of preselected, established marker genes representing the major expected cell types in human heart: cardiomyocytes (pink), fibroblasts (red), endothelial cells (yellow), vascular smooth muscle cells and pericytes (cyan), leukocyte populations (green), and neurons (blue).

Finally, we obtained a second sample from the same explanted heart to which the same nuclear isolation and sncRNAseq protocols were applied to explore the reproducibility of our observations. In FACS analysis, 11% of nuclei were PCM1^+^ (Figure 10). We focused on the cardiomyocyte-depleted fraction (PCM1^-^) of nuclei, sequencing 4,760 such nuclei which resulted in 285,564,419 reads, with a mean of 59,994 reads for a median of 491 genes per nucleus. High saturation was observed in this subset of nuclei (95%). We observed the same clusters as in the first sample, including similar proportions of nuclei with the exception that the adipocyte cluster (i e *ADIPOQ* expression) was absent from this sample (Figure 11).

## 4 Discussion

In this study, we set out to develop a protocol for robust isolation of single cells from human hearts allowing single-cell transcriptional profiling. As collection and processing of fresh heart tissue from large human cohorts is infeasible, we were particularly interested in frozen heart tissue. We applied a range of enzymatic digestion protocols which readily dissociated fresh mouse hearts but consistently resulted in limited cell yield and quality from frozen mouse and human hearts, regardless of whether the tissue had been snap frozen or treated with an RNA-stabilizing agent. Arguing that further mechanical dissociation would likely further compound cell lysis, resulting in additional debris, clustering of cell fragments, and potentially transcriptional perturbations, we instead developed a protocol for nuclear dissociation from human heart, motivated by the recent successes of such methods to allow single-cell RNA sequencing of neuronal tissues (Grindberg et al., 2013; Habib et al., 2016; Lacar et al., 2016; Lake et al., 2016) and renal tissue (Lake et al., 2019) which also cannot be readily digested into high-quality cell suspensions, especially when frozen. The mechanisms for the higher stability of cardiac nuclei to freezing remain unclear but is likely related to the higher durability of the nuclear envelope and its stabilizing nuclear lamina compared to the single-layered cell membrane. We show using expression arrays that nuclear transcriptomes are highly correlated with cytosolic and whole-cell transcriptomes of human heart cells. These findings are supported by previous studies of neuronal tissues in which transcriptomes of single cells and single nuclei have displayed similarly high concordance (Bakken et al., 2018; Lake et al., 2017). Nevertheless, some transcripts are well known to be enriched in the nucleus and so all disease associations cannot necessarily be generalized to the whole cell (Zhou and Wang, 2020). We further show that, for studies focusing on the non-cardiomyocyte components of the heart, PCM1-gating can be used to efficiently downsample the fraction of cardiomyocytes. We then applied our nuclear isolation protocol to two independent samples from a human heart and performed RNA-sequencing of single nuclei using a commercially available microdroplet-based microfluidic platform to obtain a single-cell transcriptomic perspective of a human heart.

A fundamental use of single-cell transcriptomic data is the deconvolution of highly complex transcriptomes across individual cells into distinct cell types. It is challenging to precisely define what constitutes a ‘cell type’, why the unbiased identification of cellular phenotypes from global data-driven analysis represents an important application of single-cell analysis. In our analysis, we find eight clusters that were broadly consistent across different modelling parameters, and across the two samples from the same individual. Using established markers of cell types and pathway-based clustering incorporating global transcriptional profiles we infer information on the identities of such clusters. Our findings from two left ventricular free wall samples of one heart with DCM are consistent with the view that fibroblasts, cardiomyocytes and endothelial cells represent the predominant cell types in human hearts (Bergmann et al., 2015), followed by smooth muscle cells and pericytes, macrophages, lymphocytes, neurons, and adipocytes. We note that these findings are corroborated by recently published surveys of cell types present in human hearts without heart disease based on transcriptional analysis of single-nuclei (Litviňuková et al., 2020; Tucker et al., 2020; Wang et al., 2020).

To allow the specific study of cardiomyocytes or cardiomyocyte-depleted nuclei, our results provide important validation of a PCM1-based cell sorting method. Using this method, we confirm the view that analysis of the bulk heart transcriptome is mainly correlated with the cardiomyocyte-enriched PCM1^+^ cell subset. Even though cardiomyocytes only represented 11–29% of cardiac nuclei, this observation is consistent with the large volume and high RNA content of cardiomyocytes, as also indicated by the lower RNA-sequencing saturation observed in cardiomyocytes. We note that results from the cardiomyocyte-enriched samples need to be viewed with some degree of caution, given that one quarter of human cardiomyocytes have been reported to be multinucleated (Bassaneze and Lee, 2018), potentially resulting in statistical dependence between transcriptomes of PCM1^+^ nuclei. The notion that the smaller, distinct cardiomyocyte cluster observed, characterized by natriuretic peptide expression, could represent such secondary nuclei rather than distinct cells is intriguing and warrants further exploration.

More detailed analysis of the constituents of individual nuclear clusters, importance of multinucleation in cardiomyocytes, robustness across individuals and sampling locations, and the differential expression of individual genes and pathways within cell types across healthy hearts and diverse conditions will require deeper sequencing of more cells from multiple samples and functional validation with orthogonal methods. In addition, the coupling of such data to genetic (van der Wijst et al., 2020) and epigenetic (Jia et al., 2018) information offers a particularly promising approach to obtain pathophysiological insights into heart disease. It is also worth noting that the microfluidic platform and protocol used here enables rapid, cost-effective and scalable manipulation of thousands of cells or nuclei from relatively small sample volumes with high capture efficiency, but with the limitation of relatively lower sensitivity to genes with low expression as compared to other platforms (Haque et al., 2017). The use of complementary pipelines may therefore provide additional depth of cellular phenotyping to that presented here although with lower throughput.

## 5 Conclusions

In comprehensively evaluating protocols for isolation of individual cells from human and mouse hearts, we confirm the validity of single-nucleus but not single-cell isolation for frozen heart tissue. Furthermore, we show that PCM1-gating can be used for cardiomyocyte depletion with high efficiency, and provide a perspective of the cell types present in an adult human heart. When applied in scale to frozen human hearts, we anticipate that the methods described and validated here will contribute greatly to enhance understanding of cardiac pathobiology on the single cell level.

## Declarations

### Author contribution statement

Neha Pimpalwar: Performed the experiments; Wrote the paper.

Tomasz Czuba: Analyzed and interpreted the data; Wrote the paper.

Maya Landenhed Smith, Johan Nilsson: Contributed reagents, materials, analysis tools or data; Wrote the paper.

Olof Gidlöf: Conceived and designed the experiments; Performed the experiments; Analyzed and interpreted the data; Contributed reagents, materials, analysis tools or data; Wrote the paper.

J. Gustav Smith: Conceived and designed the experiments; Analyzed and interpreted the data; Contributed reagents, materials, analysis tools or data; Wrote the paper.

### Funding statement

J. G. Smith was supported by the 10.13039/501100003793Swedish Heart-Lung Foundation (2016-0134, 2016-0315 and 2019-0526), the 10.13039/501100004359Swedish Research Council (2017-02554), the 10.13039/501100004359European Research Council (ERC-STG-2015-679242), the 10.13039/501100003173Crafoord Foundation, 10.13039/501100011077Skåne University Hospital, the Scania county, governmental funding of clinical research within the Swedish National Health Service, the 10.13039/501100004063Knut and Alice Wallenberg foundation, the Swedish Research Council (Linnaeus grant Dnr 349-2006-237, Strategic Research Area Exodiab Dnr 2009-1039) and Swedish Foundation for Strategic Research (Dnr IRC15-0067). O. Gidlöf was supported by the 10.13039/501100003793Swedish Heart-Lung Foundation (2019-0409, 2018-0335 and 2017-0622), the 10.13039/501100003173Crafoord Foundation, the 10.13039/501100006285Magnus Bergvall Foundation and the 10.13039/501100005753Royal Physiographic Society.

### Data availability statement

Data associated with this study has been deposited at NCBI Gene Expression Omnibus under the accession number GSE161067 for the microarray dataset and GSE161153 for the single nucleus RNA-sequence dataset.

### Declaration of interests statement

The authors declare no conflict of interest.

### Additional information

No additional information is available for this paper.

## Appendix A Supplementary data

Supplementary_Material.docx Supplementary_Material.docx
