# Supplementary material for: Methods for isolation and transcriptional profiling of individual cells from the human heart
Source: Heliyon. 2020 Dec 29;6(12):e05810. doi: 10.1016/j.heliyon.2020.e05810 (PMC7779736; doi:10.1016/j.heliyon.2020.e05810)
Supplement: Supplementary_Material.docx [file mmc1.docx]

**FIGURE S1. Concordance of nuclear with cytoplasmic and whole-cell transcriptomes.**


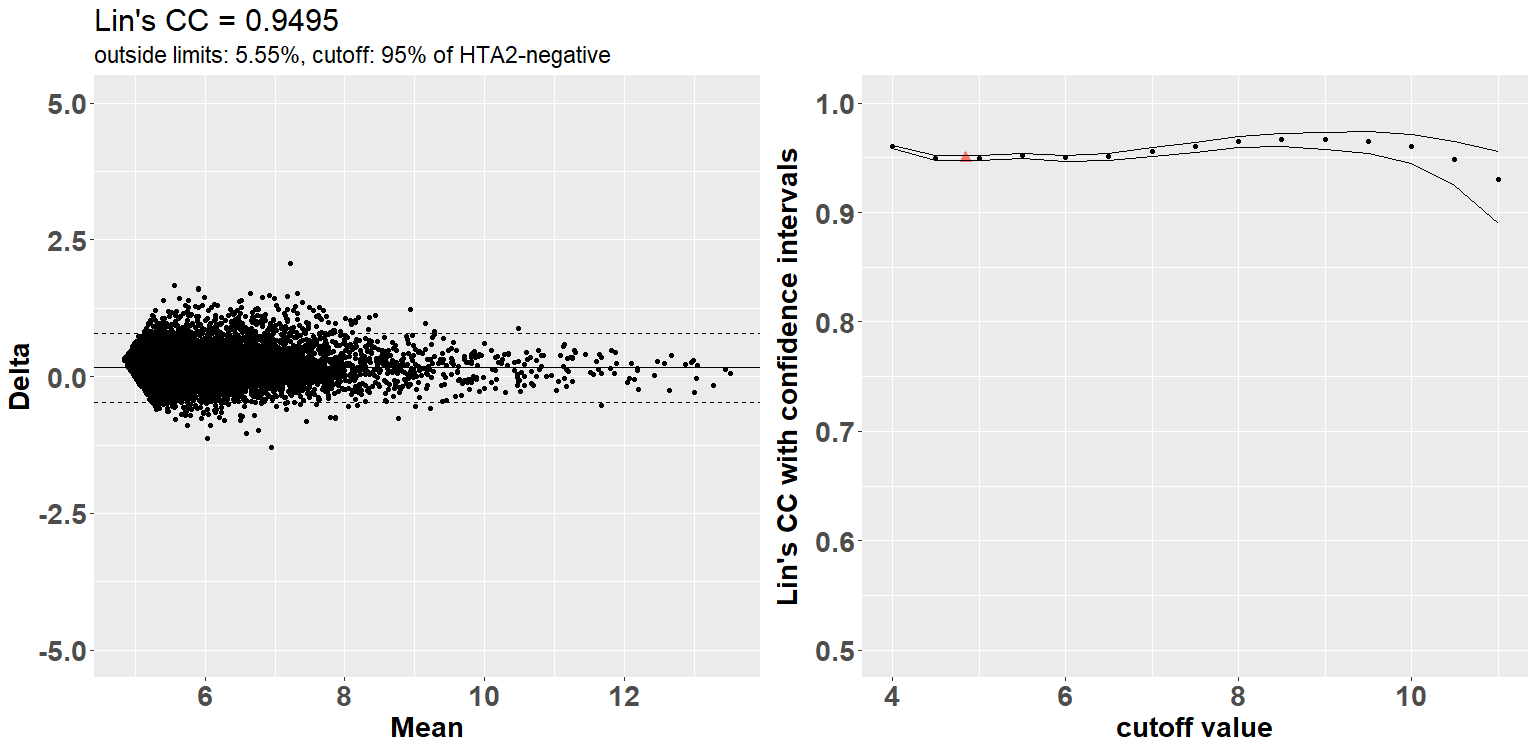

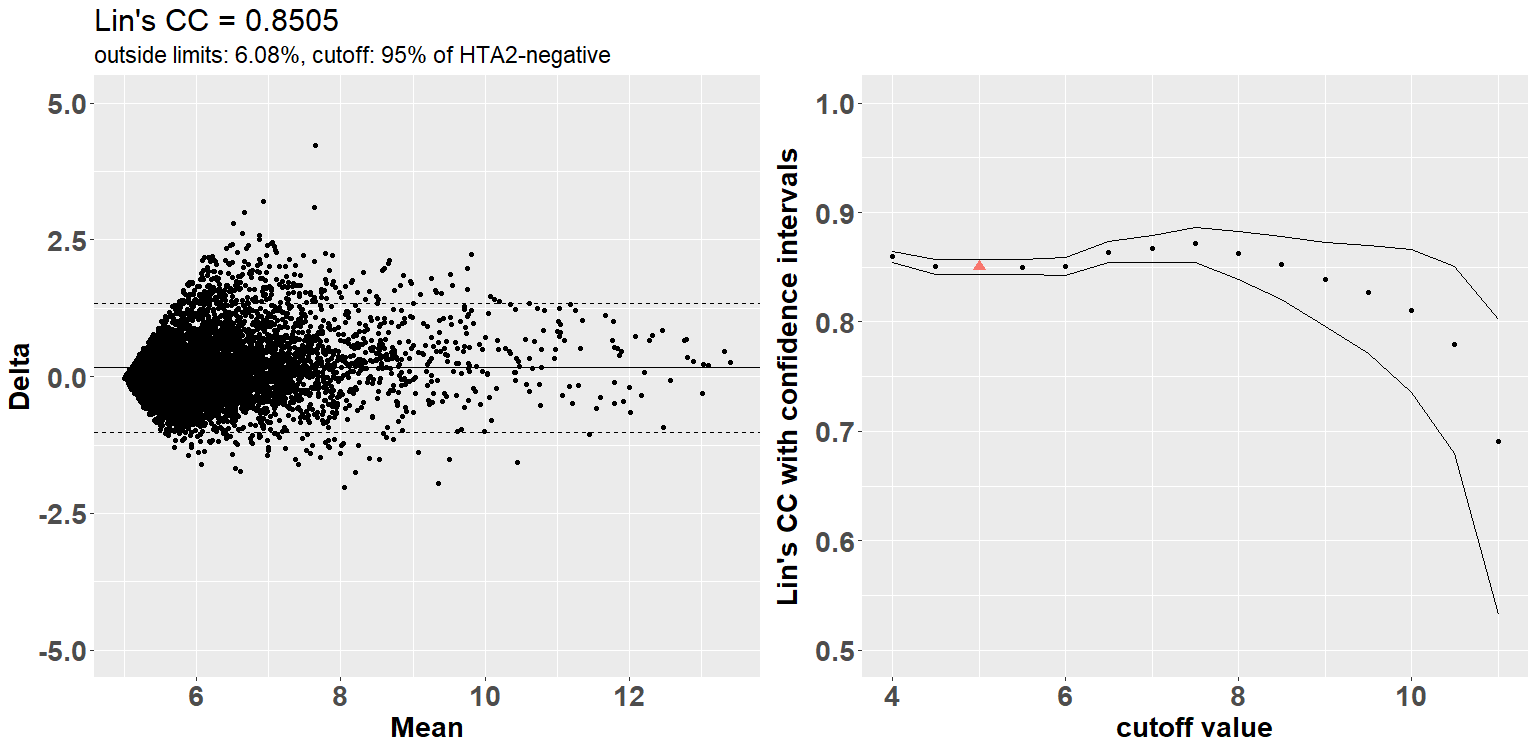

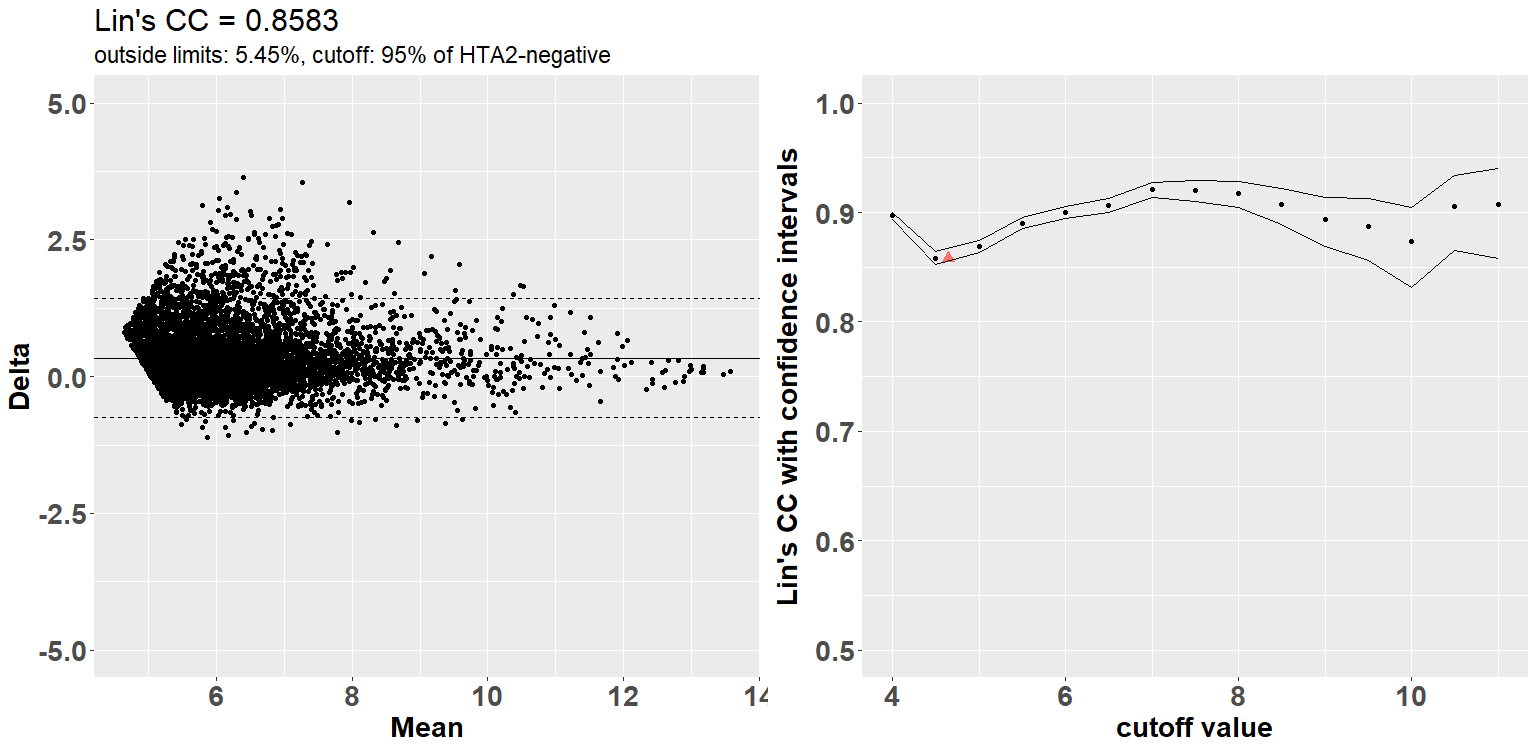

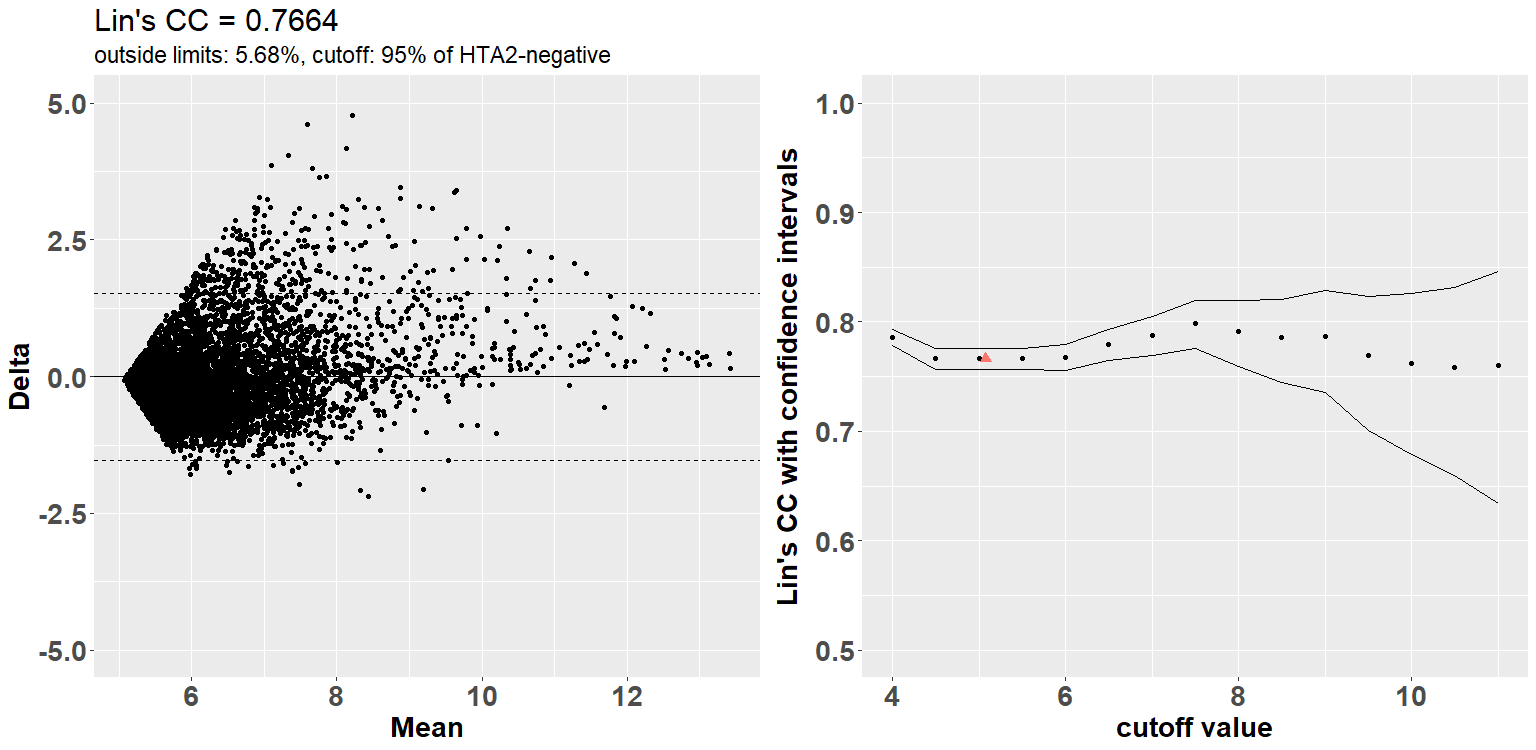

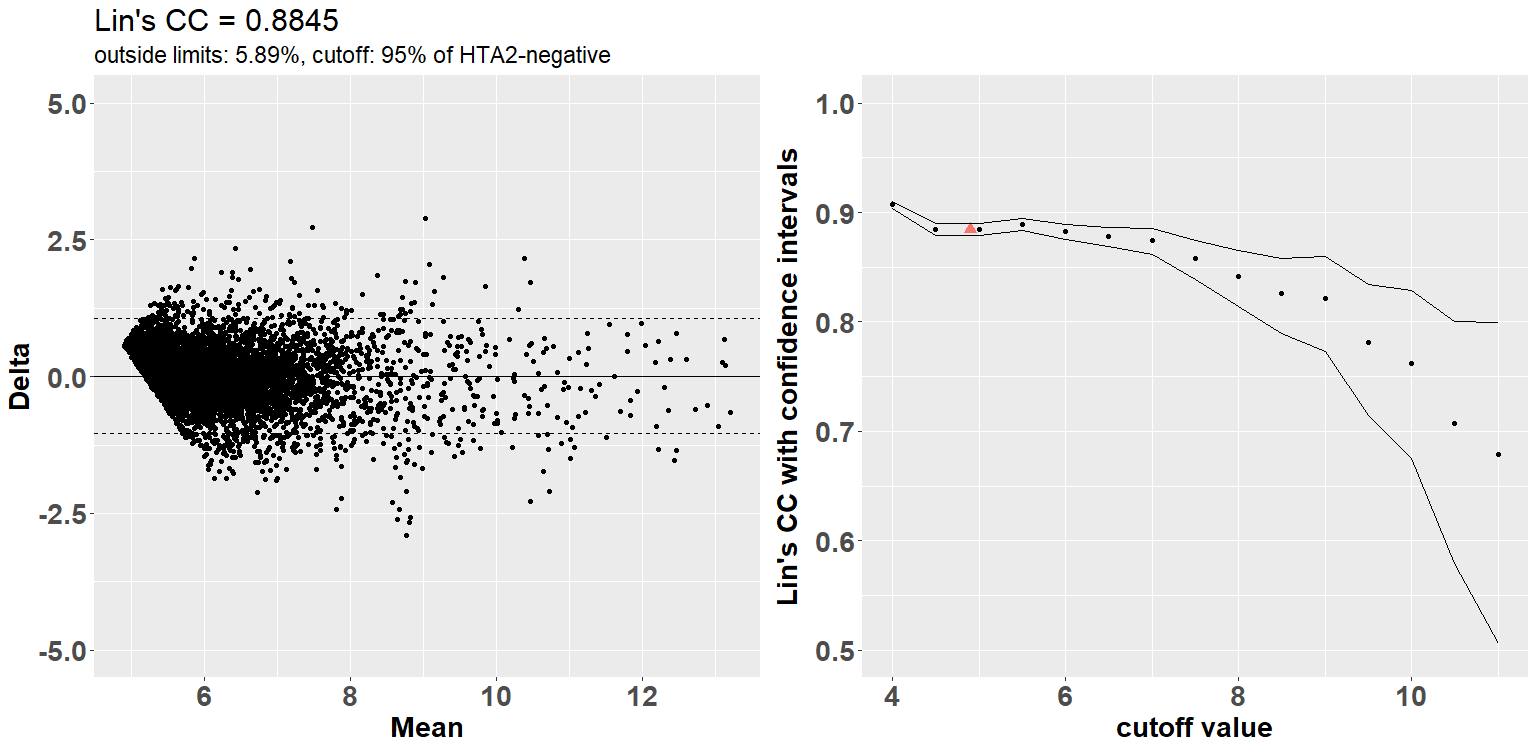

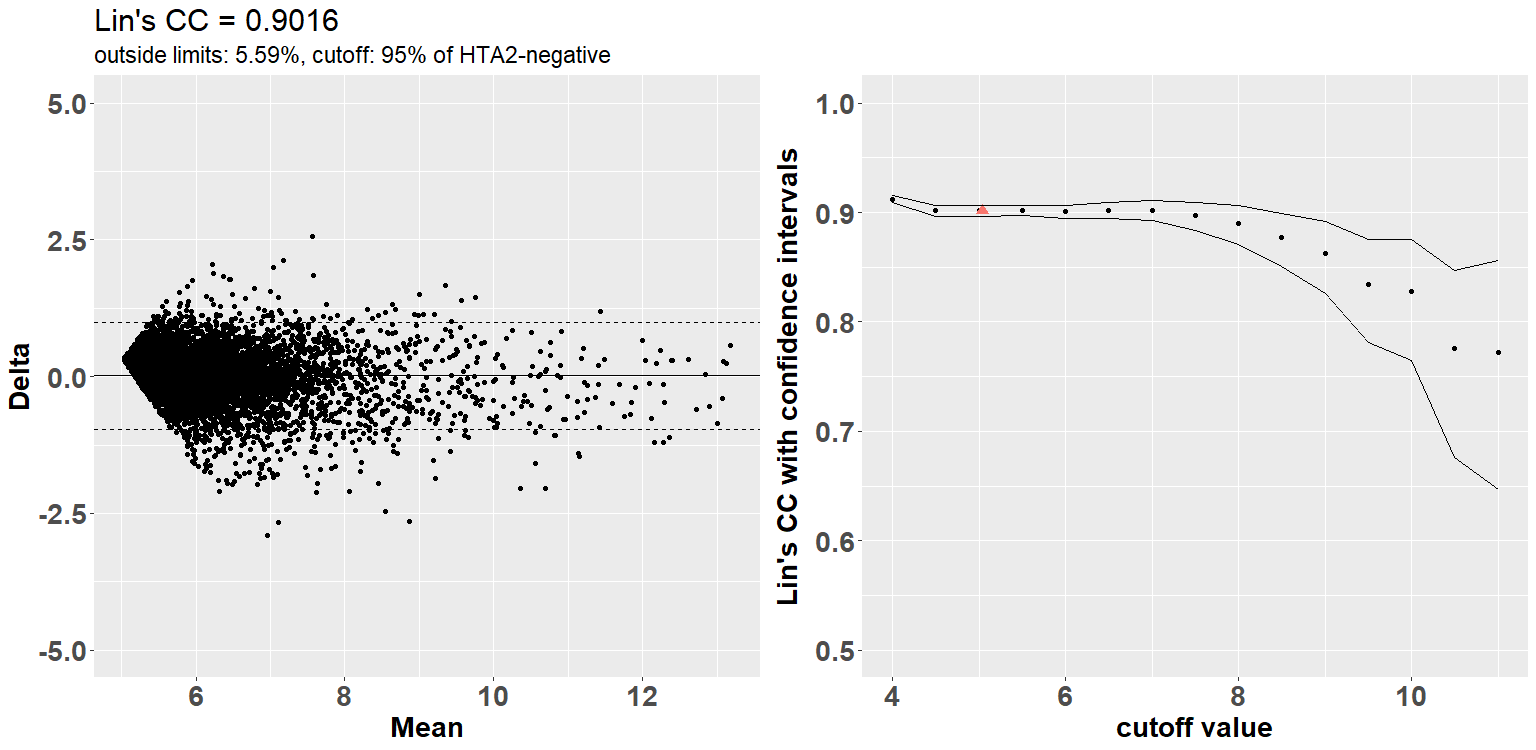


A

B

C

D

E

F

Bland-Altman plots and Lin’s concordance coefficients with 95% confidence intervals for nuclear transcriptomes with cytoplasmic and whole-cell transcriptomes in induced pluripotent stem cell derived cardiomyocytes (cytoplasmic, A; whole-cell, B), primary human cardiac fibroblasts (cytoplasmic, C; whole-cell, D), and cardiac microvascular endothelial cells (cytoplasmic, E; whole-cell, F). Bland-Altman plots indicate the absolute difference in expression between transcriptomes across different levels of mean expression. X-axis for concordance plots indicates different thresholds for non-expressed transcripts representing technical noise. The red triangle in the concordance plots indicate the cutoff value used in Bland-Altman plots (95 percentile of the averaged negative control probe signal).

**FIGURE S2. Correlation of cardiomyocyte-enriched and -depleted heart tissue fractions with bulk heart tissue expression and stem cell-derived cardiomyocytes in culture.**


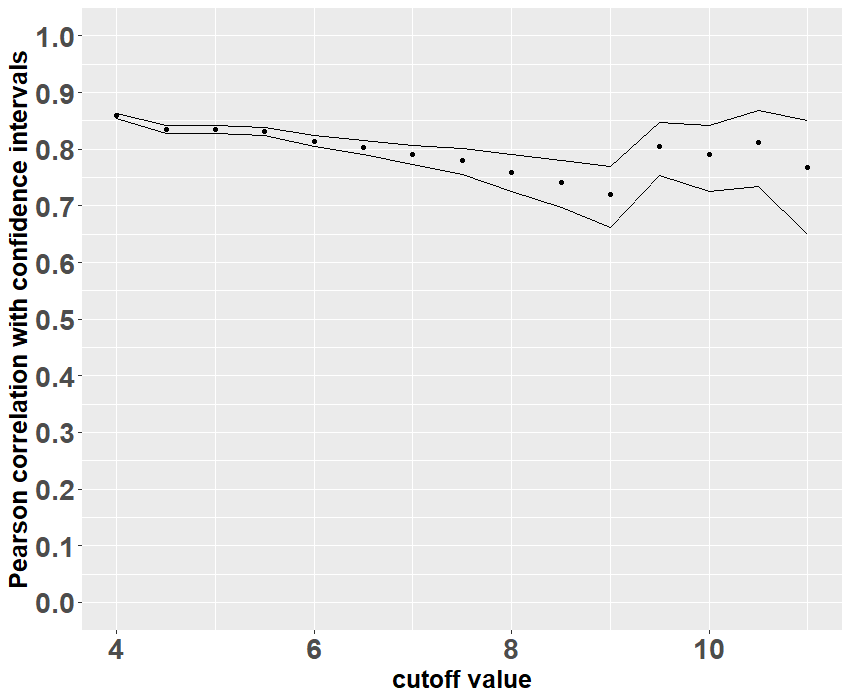


A


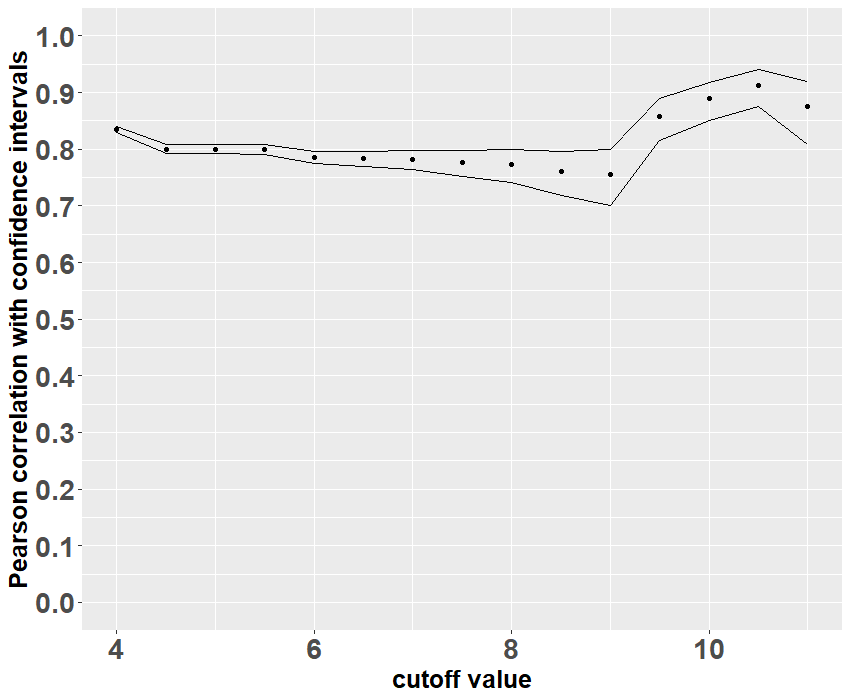


B


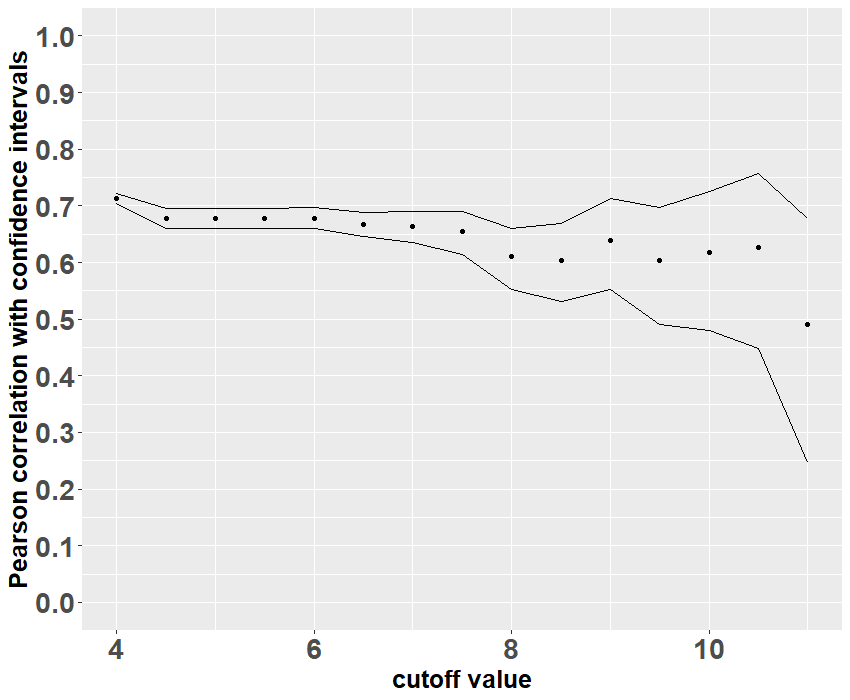


D


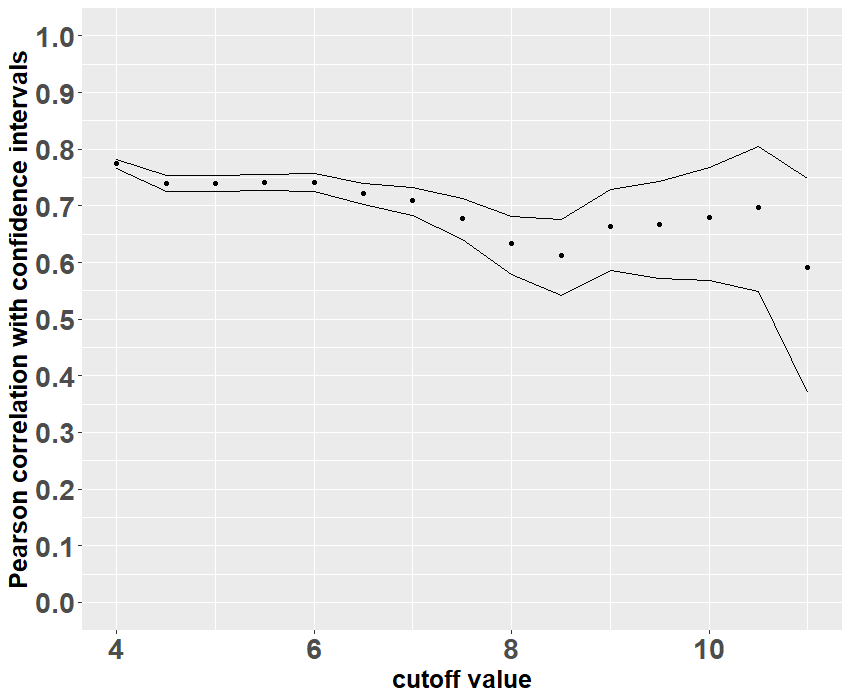


C

Pearson’s correlation coefficients with 95% confidence intervals for PCM1^+^ sorted nuclei with bulk heart transcriptomes (A) and induced pluripotent stem cell-derived cardiomyocytes (B). For PCM1^-^ sorted nuclei with bulk heart transcriptomes (C) and induced pluripotent stem cell-derived cardiomyocytes (D). X-axis indicates different tresholds for non-expressed transcripts representing technical noise.

**FIGURE S3. Analysis of intact nucle with brightfield microscopy.**

**
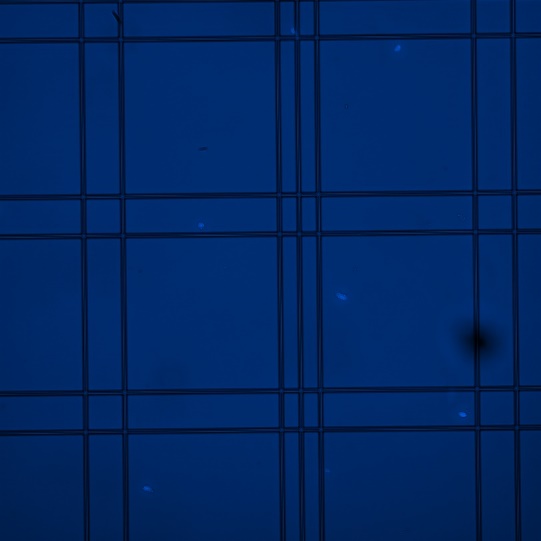

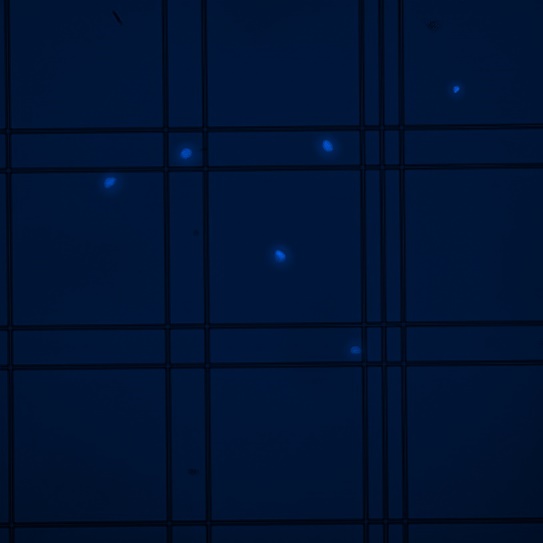
**

Brightfield microscopy images of isolated nuclei stained with Hoechst. Left, PCM1^-^ nuclei. Right, PCM1^+^ nuclei.

**FIGURE S4. Effect of clustering parameters on identified single-nucleus clusters.**


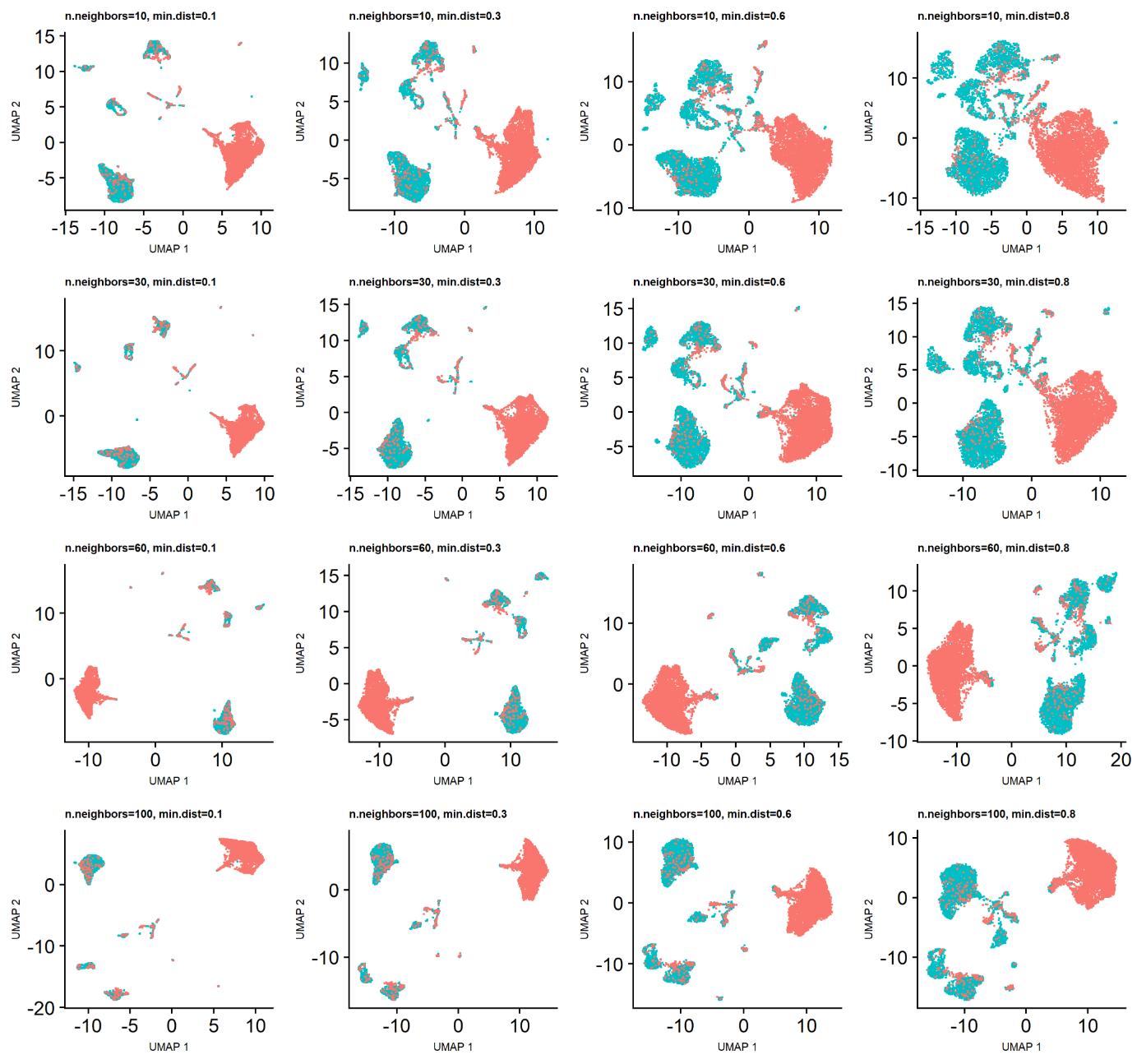


Uniform Manifold Approximation and Projection (UMAP) projections based on varying values for the two key UMAP parameters: numbers of neighbors (n.neighbors) between 10, 30, 60 and 100, and the minimum distance (min.dist) between 0.1, 0.3, 0.6 and 0.8.

**FIGURE S5. Expression of cell type informative markers across single-nucleus clusters.**

**
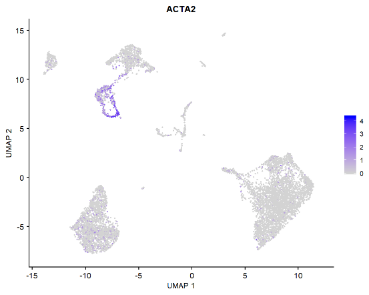

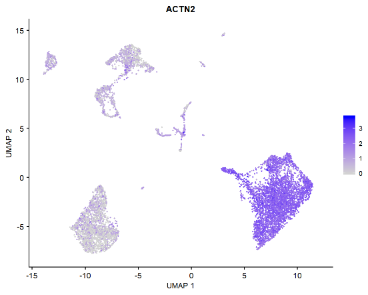

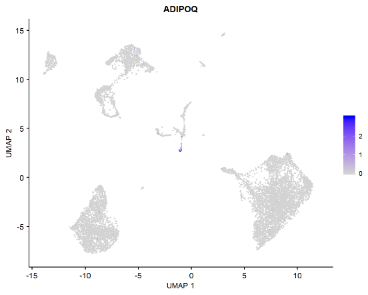

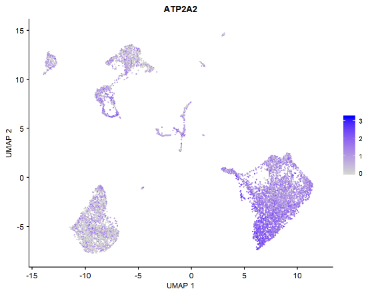

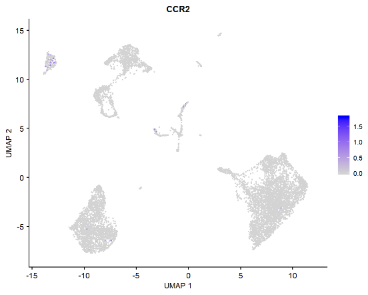

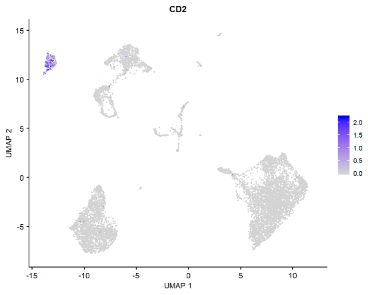

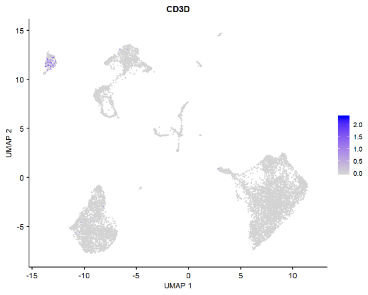

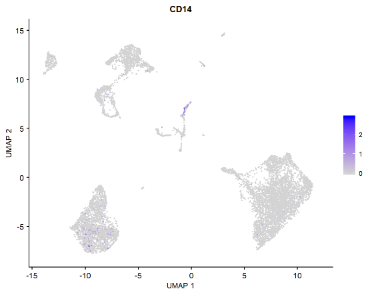

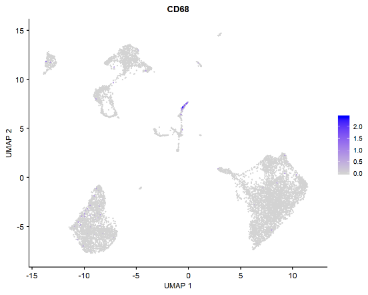

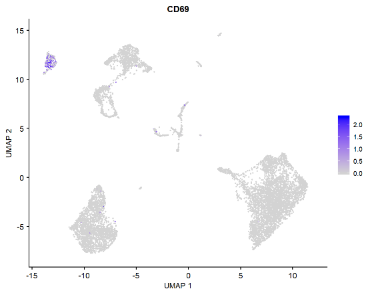

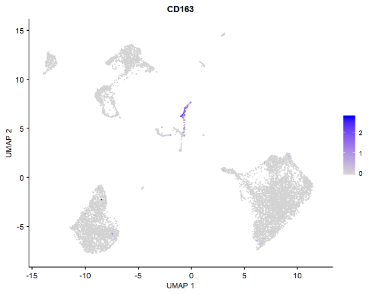

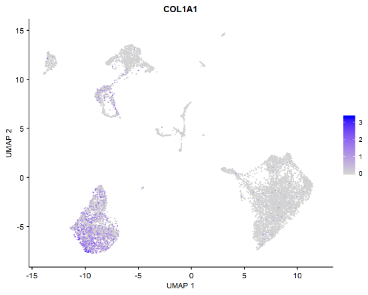

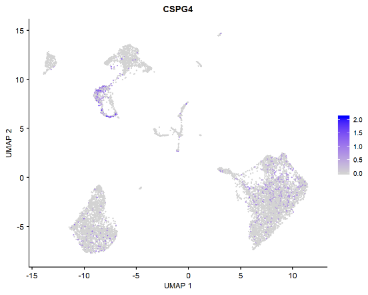

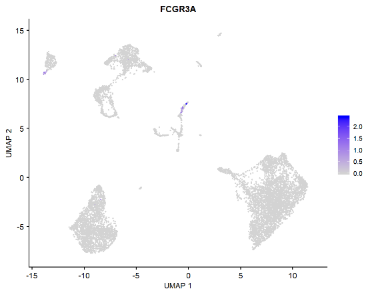

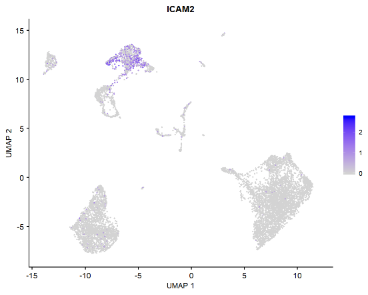

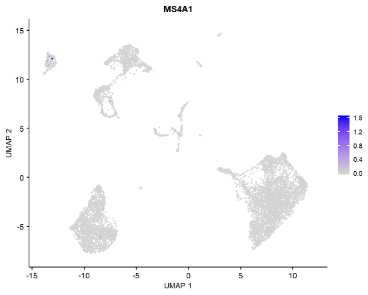

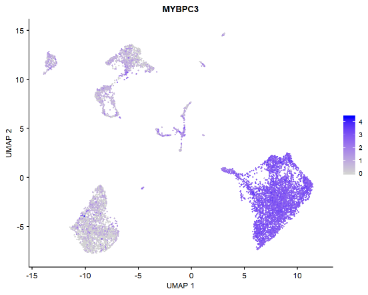

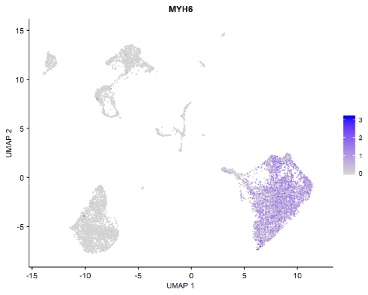

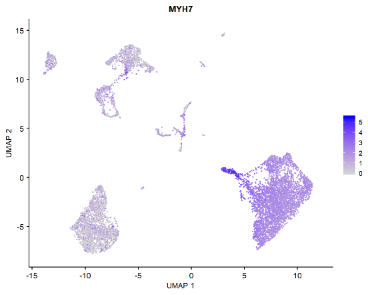

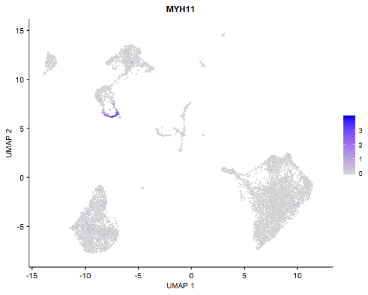

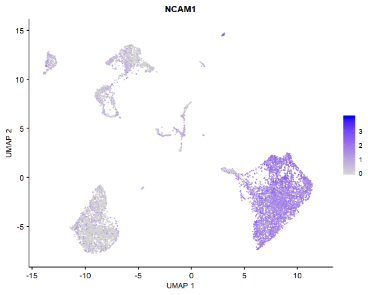

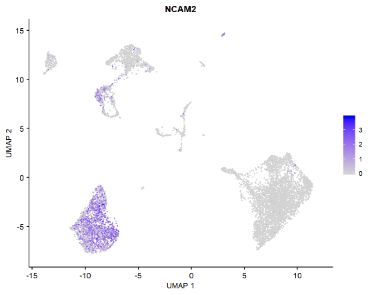

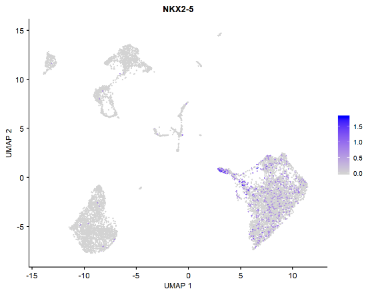

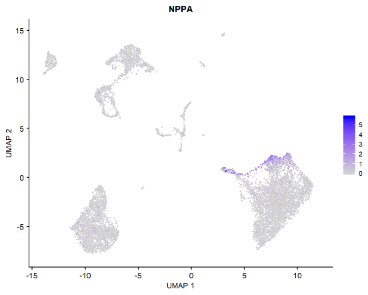

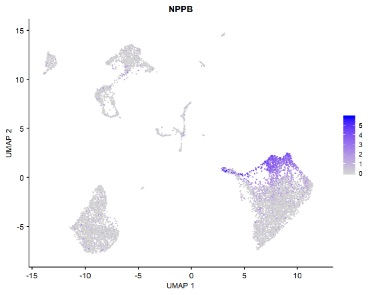

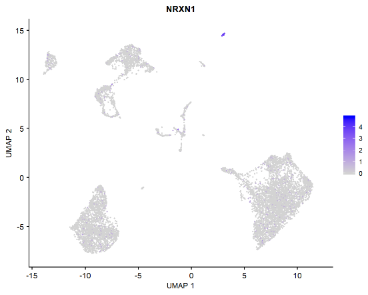

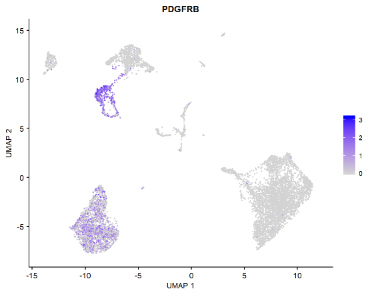

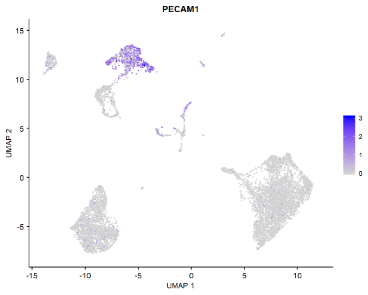

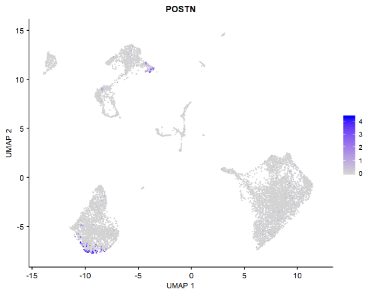

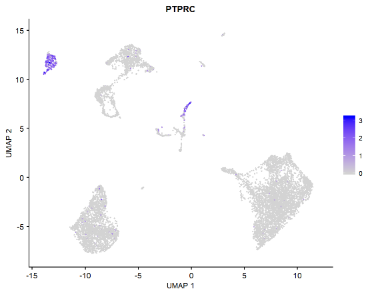

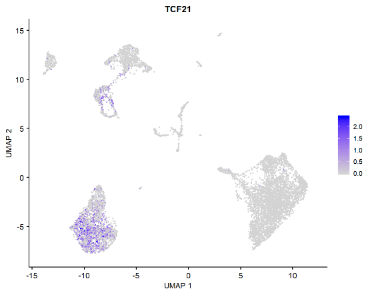

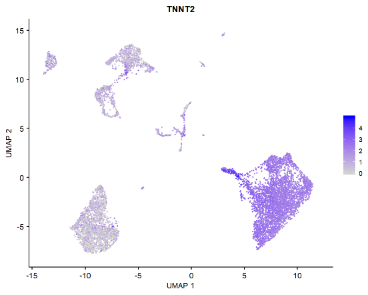

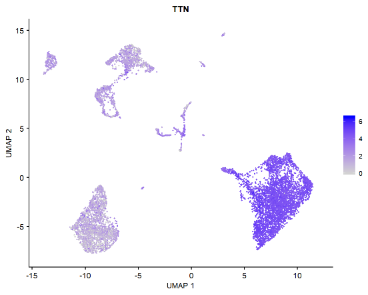

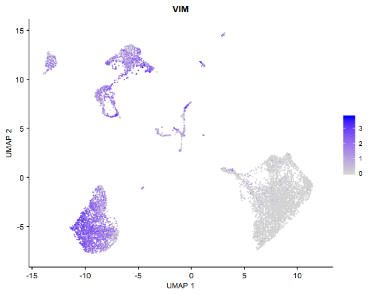

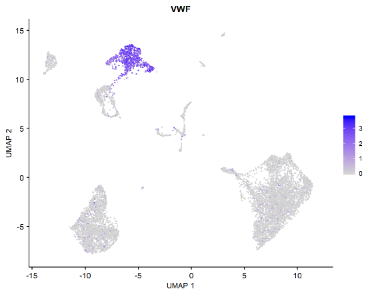
**

Uniform Manifold Approximation and Projection (UMAP) plots with expression of each of the cell type informative marker genes indicated.
